# A theory-based practical solution to correct for sex-differential participation bias

**DOI:** 10.1186/s13059-022-02703-0

**Published:** 2022-06-27

**Authors:** Hanbin Lee, Buhm Han

**Affiliations:** 1 Department of Medicine, Seoul National University College of Medicine, 103 Daehak-ro, Jongno-gu, Seoul, 03080 Republic of Korea; 2 Department of Biomedical Sciences, BK21 Plus Biomedical Science Project, Seoul National University College of Medicine, 103 Daehak-ro, Jongno-gu, Seoul, 03080 Republic of Korea; 3 Interdisciplinary Program in Bioengineering, Seoul National University, Seoul, 08826 Republic of Korea

## Abstract

Most genomic cohorts are retrospective where the exposures and outcomes are predetermined prior to sample collection. Therefore, a spurious association between an exposure and an outcome can arise if both variables affect study participation. Such concerns were raised in previous studies questioning the representativeness of the UK Biobank. Recently, a genome-wide association study (GWAS) on biological sex found many autosomal hits and non-negligible autosomal heritability which the authors attribute to selection bias. In this study, we propose a simple and a practical method that can overcome sex-driven selection bias based on theoretical analysis and simulations.

**Supplementary Information:**

The online version contains supplementary material available at 10.1186/s13059-022-02703-0.

## Background

The study design of large genomic cohorts is usually retrospective, which means that the samples are collected after the exposures and the outcomes are determined. In such designs, participation bias may be present because the possibility of both outcomes and exposures affecting study participation cannot be excluded by time ordering as in prospective studies. One notable example of retrospective genomic cohorts is the UK Biobank [1]. Fry and colleagues found that the participants of the UK Biobank are healthier and wealthier than the general population of the UK [2]. A survey by Huang reported that performing genetic analysis upon such selective samples can lead to substantial bias [3]. A recent genome-wide association study (GWAS) on biological sex has shown that autosomal variants are associated to biological sex, which the authors attributed to selection bias [4]. Through simulations, the authors further demonstrated the potential impact of sex-driven study participation (*sex-differential participation* in their terminology) on downstream analysis.

Although sex-differential participation can affect the downstream GWAS analysis of many traits, no method has been proposed that can systematically correct for this bias. Inverse probability weighting (IPW) is a famous approach that is used to correct for selection bias [5, 6], but this method has little utility in a genomic context because the genotypes of non-participants are not observed. Pirastu et al. suggested solutions that require census allele frequencies of the target population [4]. Still, such information may not be readily available.

In this study, we propose a simple and a practical method that can overcome sex-driven selection bias based on theoretical analyses and simulations. We first build a general theoretical foundation of selection bias. This theory contains the suggested models by Pirastu et al. [4] as special cases and can provide predictions and guidance on design of future genomic cohorts. Based on theoretical speculation, we found that study participation rate is a key driving factor that determines the strength of selection bias. Under low participation rate, it can be shown that sex-driven selection bias is negligible in sex-stratified GWAS. This importantly implies that sex-stratified GWAS followed by fixed-effects meta-analysis is robust to sex-driven selection bias. We demonstrate that this simple solution is effective against bias by extensive simulations.

## Results and discussions

The *selection bias* (or collider bias) is a non-causal spurious association between two variables conditional on their common effect [5]. In epidemiologic studies, it is known that selection bias can lead to awkward conclusions. For example, a classic study by Berkson [7] found a negative association between cholecystitis and diabetes based on in-hospital patients. This phenomenon called the Berkson’s paradox, which became a famous idiom for selection bias, happens because both cholecystitis and diabetes increase the risk of hospitalization. Hence, looking only at in-hospital patients creates a non-causal association between them.

A notable example in a genetics study was reported by Day and colleagues [8]. They showed that biological autosomal variants are robustly associated to sex after adjusting for height in the regression. Pirastu and colleagues found that such associations are present even without covariate adjustment in the UK Biobank and 23andMe database [4]. Dudbridge and colleagues proposed a correction for collider bias that occurs due to the regression adjustments [9]. Extending this approach, Barry and colleagues proposed a correction method for one-sample Mendelian randomization [10]. These methods are not applicable to the current problem of sex-driven selection bias, because the mode of conditioning on a collider is different from regression adjustments. The two papers account for conditioning based on linear regression models but such parametric strategy cannot be generalized to conditioning on study participation. Hence, neither a method nor a model that quantitatively evaluates the magnitude of collider bias under selective study participation yet exists.

In this article, we propose a theoretical foundation that includes the findings of Pirastu et al. [4] as special cases and validate their observations in the light of our theory. We describe a detailed theoretical model in Additional File 1: Supplementary Note. Importantly, our theory suggests a simple and practical solution to correct for the sex-differential participation bias in an association study. Moreover, our theory makes predictions about the fate of the bias in the future as the participation rate grows, which will serve as a basis for future study designs. Here, we use the term sex-independent if the effect of a trait or a variant on study participation is equal in both sexes (no sex-gene or sex-trait interaction exists) and the term sex-differential if they are not the same (such interaction does exist).

**Fig. 1:**
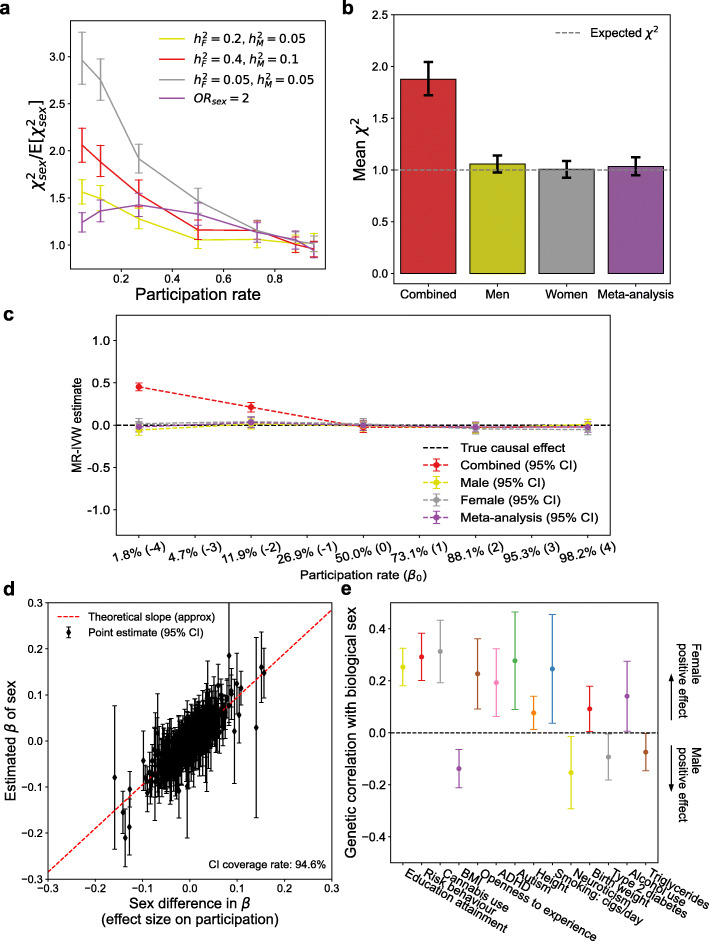
The underlying meaning of sex GWAS. **a** Observed mean chi-squared statistics over expected chi-squared statistics of sex GWAS with respect to differing participation rates under sex-differential and sex-independent GWAS. Different lines correspond to different liability scale heritabilities of study participation for each sex. **b** Observed mean chi-squared statistics under sex-differential participation of sex-combined GWAS, sex-stratified GWAS, and fixed-effects meta-analysis of sex-stratified GWAS. **c** MR-IVW (inverse variance weighted) estimates of two independent binary traits using summary statistics from sex-combined and sex-stratified GWAS. **d** Difference between marginal effect size of a locus on study participation is the determinant of sex GWAS under study participation. The slope is the difference of study participation rate between male and female. **e** Genetic correlation of complex traits with biological sex in the UK Biobank. The estimates were adopted from Pirastu et al. [4]

First, our theoretical model suggests that the participation rate (π) is a key driving factor of participation bias. Indeed, even with the other simulation parameters fixed, the strength and the direction of collider bias differ substantially by participation rate as shown in Fig. 1a. Although Pirastu et al. explored the effect size of potential determinants of study participation (Supplementary Figure 2 of Pirastu et al. [4]), without considering the participation rate, conclusions regarding the trend of bias with respect to the magnitudes of simulation parameters will only be partial. Our model provides a theoretical justification for why sex-independent participation cannot explain the observed heritability in the UK Biobank and 23andMe. As the bias is proportional to the between-sex participation rate difference (1.3% in the UK Biobank [2]) according to the theory, the bias under sex-independent participation cannot reach the observed value (Additional File 1: Supplementary Note).

Second, our theory provides a practical solution to correct for the bias in an association study. The theory predicts that sex-stratified GWAS is nearly immune to sex-differential participation bias (Additional File 1: Supplementary Note) conditioned on a low participation rate. Our simulations confirm this theory and show that the bias is predominant only in sex-combined GWAS (Fig. 1b and Additional File 1: Figure S1-3). Therefore, in most situations with low participation rate, one can correct for the sex-differential participation bias simply by performing sex-stratified GWAS and meta-analyzing them. This proves why the genetic correlation between male and female-only GWAS was mostly unaffected by sex-differential participation as shown in a simulation of Pirastu et al. (Supplementary Figure 5 of Pirastu et al. [4]). The results extend to Mendelian randomization where sex-stratified and meta-analyzed summary statistics are less likely to produce bias (Fig. 1c and Additional File 1: Figure S4-6). We show that this stratified approach does not result in loss of statistical power compared to combined GWAS in a representative sample without participation bias (Additional File 1: Figure S7).

Third, the theory (Theorem 2 in Additional File 1: Supplementary Note) suggests that the observed effect size of a genetic variant on biological sex is mainly driven by (and is proportional to) the between-sex effect size difference of the variant on study participation. Our simulations confirm this relationship (Fig. 1d). Thus, assessing the genetic correlation of a trait to sex provides a quantitative measure of sex-differential participation: a strong genetic correlation of a trait with sex means that the effect size distribution aligns with the between-sex effect size difference on study participation, where a positive correlation corresponds to the larger positive effects in female than male (Fig. 1e and Additional File 1: Supplementary Note).

Fourth, our theory predicts the fate of different types of participation biases as the participation rate grows (Fig. 1a). While sex-independent bias is relatively small across varying participation rates, sex-differential bias can be very large when the participation rate is low. Hence, when recruiting a fixed number of participants, collecting as many samples from a fixed target population as possible to increase the participation rate is recommended. Unclear target population definition can reduce the participation rate even if the number of participants increases due to the expansion of the implicit underlying target population. For example, the UK Biobank contacted the UK residents of age 40–69 in 2010. On the other hand, it is nearly impossible to know who the potential customer in case of commercial cohorts like 23andMe is. In the latter case, participation rate is extremely difficult to estimate as the denominator (the number of potential customers) is ill-defined.

## Conclusions

In this study, we showed that the strength of sex-driven selection bias becomes nearly zero if the data were analyzed in a sex-stratified manner under low participation rate. Thus, one can perform a reliable GWAS and downstream analysis under current study designs by simply conducting sex-stratified analysis. Our suggestion is practical and easy as it does not require any additional method or preprocessing. Notably, the suggestion is grounded by theory with proofs, hence reliable. This is a simple but useful finding that was missed by previous studies that were based solely on simulations.

The theory is general enough to incorporate population-based studies like the UK Biobank and electronic health record (EHR)-based studies like DiscovEHR [11]. This is because the mathematics behind the theory does not require any assumptions related to the specific mode of participation into the study. Unless the logit model of participation is not violated, it is likely that our theory will hold regardless of the study design.

However, our theory also predicts that this solution is not likely to be applicable to future studies with higher participation rate. Instead, it provides guides for such future studies that can minimize sex-driven selection bias. Also, even when perfect correction is not attainable, the theory gives a bound around the obtained estimate where the true effect lies.

Our framework also provides insight into other forms of collider bias. The theory does not rely on a particular characteristic of sex except that it is binary so the argument can be applied to any binary variables that affect study participation. Furthermore, the proof can be naturally extended to any discrete variables with more than two values. In such a situation, given a low participation rate, the bias is likely to be small unless a genetic variant has an interaction with a variable that affects study participation, as shown in an earlier simulation study [6]. If interaction exists, we can use a similar stratifying strategy for the multi-value variable to remove potential bias. In the future, further studies dealing with continuous variables are warranted [12], because continuous variables can also affect study participation.

We expect our work to serve as a theoretical milestone for future methodological development and study designs that aim to account for participation bias in large genomic cohorts.

## Methods

The detailed method and the proofs can be found in the Additional File 1: Supplementary Note.

### Peer review information

Andrew Cosgrove was the primary editor of this article and managed its editorial process and peer review in collaboration with the rest of the editorial team.

### Review history

The review history is available as Additional file 2.

## Supplementary Information

**Additional file 1.** All supplementary information.

**Additional file 2.** The review history.

## Data Availability

The code used to produce the results in the paper can be found at GitHub (https://github.com/hanbin973/autosomalsexgwas) and the associated Zenodo (https://zenodo.org/record/6426487) [13]. The code is freely available under an open source license (GPL-3.0). The genetic correlation summary statistics of the UKB data was retrieved from the supplementary materials of Pirastu et al. [4]. No raw material from UKB was used in this paper.
