# Supplementary material for: A theory-based practical solution to correct for sex-differential participation bias
Source: Genome Biol. 2022 Jun 27;23:138. doi: 10.1186/s13059-022-02703-0 (PMC9238114; doi:10.1186/s13059-022-02703-0)
Supplement: Supplementary file 1 — Additional file 1. All supplementary information. [file 13059_2022_2703_MOESM1_ESM.pdf]

Supplementary Note

Hanbin Lee

March 24, 2022

Contents

1 Introduction **2**

2 Three models of collider bias **3**

3 An analytic expression for collider bias **4**

4 The sex-gene interaction model **6**

5 The effect of sex-differential participation on the genotype-trait associations of other traits **8**

6 Sex-stratified analysis can substantially reduce the bias caused by sex-differential participation on the associations of other traits **12**

7 Accounting for sex-biased population prevalence **14**

8 Appendix: Methods **15**

8.1 Trait simulation . . . 15
8.2 Sex GWAS χ^2 versus participation rate experiment . . . 15
8.3 Robustness of sex-stratified GWAS . . . 15
8.4 Bias in MR-IVW versus study participation rate experiment . . . 15
8.5 β difference between sex and sex GWAS . . . 15

1 Introduction

This section introduces the basic concepts that are required to understand the current study. C is said to be a *collider* of A and B if it is a common effect of A and B. As we are particularly interested in a case where A and B are marginally independent, the relationship between A, B and C is summarzed in the following directed acyclic graph.

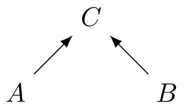

The particular variables of interest in this study are biological sex (defined by the sex chromosome configuration), genetic variants and study participation. We write $Z \in \{0, 1\}$ for sex, $G_j \in \{0, 1, 2\}$ for the j-th genotype and $C \in \{0, 1\}$ for study participation. Let $G = \{G_j | j = 1, ...\}$.

This translates the diagram above into the following.

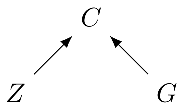

Although Z and G are marginally independent ($Z \perp\!\!\!\perp G$), they may be conditionally dependent when conditioned on C, i.e. $Z \not\perp\!\!\!\perp G|C$ [1]. This conditioning is represented as a box surrounding C. This spurious dependence between Z and G conditional on C is called the *collider bias*.

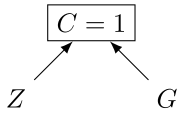

As we only observe samples who participated in a study, the diagram above predicts that spurious dependence between Z and G may be observed in large genetic cohorts. Indeed, Pirastu et al. reported that such bias is present in the 23andMe and the UK Biobank datasets [2].

Additional components, for example, such as an unobserved confounder U of C and G can be included.

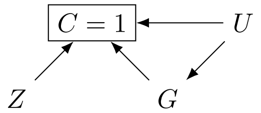

This also opens a backdoor between Z and G through the path $G \leftarrow U \rightarrow C \leftarrow Z$ conditional on C. However, this backdoor is closed regardless of conditioning on U if one does not condition on C. Hence, U will not play a significant role in the remaining arguments in this study.

2 Three models of collider bias

Pirastu et al. proposed three models that can potentially explain the association between sex and autosomal variants [2]. For completeness, we restate the three models in this section. The figures below were adopted from Pirastu et al. with some changes in the notation.

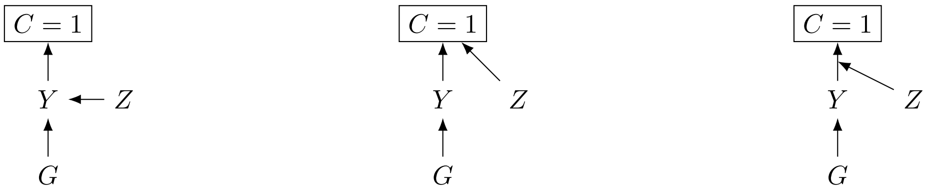

(a) Model A (b) Model B (c) Model C

Model A depicts the scenario where sex Z and a genetic variant G affects Y, where Y is a phenotype that has a causal effect on the participation C. Because C is a descendant of Y which is a collider of Z and G, conditioning on C will induce a collider bias between Z and G [1]. Model B is similar to Model A but Z influences C directly rather than indirectly through Y. Pirastu et al. used the same functional relationship of Z and G on C to depict both Model A and Model B. Specifically, for both models, they assumed the logit of the probability of $C = 1$ as a linear function of Z and G. It can be found in their supplementary material that

$$Y = G + Z + \epsilon$$
$$P(C = 1) = \frac{1}{1 + e^{-Y}}$$

for Model A and

$$Y = G + \epsilon$$
$$X = Y + Z + \epsilon$$
$$P(C = 1) = \frac{1}{1 + e^{-X}}$$

for Model B after dropping the coefficients of the predictors for notational convenience. Therefore, the association between Z and G due to the collider bias is essentially identical in both models.

In the scenario of Model C, Z alters the effect of Y on C. For example, the effect of trait Y on C can be positive in men but negative in women. This model requires a more thorough inspection. Importantly, the third figure depicting Model C is not a standard DAG. In a standard DAG, an arrow only enters and exits a node but not another arrow. However, in Model C, the arrow exiting Z enters the arrow from Y to C. This choice by Pirastu et al. comes from the fact that the standard DAG cannot explicitly express interaction between variables. A conventional way to express interaction will be using a standard DAG which is identical to Model B but stating a explicit functional form of C with G and Z as a predictor. In their supplementary material, they used a constant K to depict the modifying effect of Z on Y's effect as

$$Y = G + \epsilon$$
$$P(C = 1|Z = 0) = \frac{1}{1 + e^{-Y}}$$
$$P(C = 1|Z = 1) = \frac{1}{1 + e^{-KY}}$$

In the following chapters, we develop a general model that encompasses all three models as a special case. We will use standard DAGs to depict the situation and will attach a functional form for each variable if necessary to avoid the use of a non-standard DAG.

Finally, at this point, one might have noticed that Model A differs from Model B in that sex Z affects trait Y. For example, if Model A is true, the prevalence of trait Y can differ depending on sex, which is not the case in Model B. Although this is not an issue for the following two chapters, we will address this problem in the last section when it comes to effect.

3 An analytic expression for collider bias

In this section, we assume that study participation takes place according to the following logit model:

$$C \sim \mathrm{B}(1, \pi)$$
$$\log\left(\frac{\pi}{1-\pi}\right) = \beta_0 + \beta_{g\to c}G_j + \beta_{z\to c}Z + \epsilon$$

$\mathrm{B}(n, p)$ is a binomial distribution with n trials with probability p, G_j is the standardized genotype of the j-th variant, Z is the sex, and ϵ is the residual error following a normal distribution with mean zero and variance σ^2. β are the corresponding regression coefficients; $\beta_{g\to c}$ is the effect of G_j toward participation, and $\beta_{z\to c}$ is the effect of Z toward participation. This simple model does not assume the sex-gene interaction (sex-differential participation), but we will later extend this model to include interaction.

Let $\widetilde{\beta}_{g\to z}$ be the effect of G_j on Z caused by the collider bias. A tilde on a symbol, e.g. $\widetilde{\beta}$, stands for the biased parameter that is estimated under non-random selection into the study. Let $\pi_{c|z} = \mathrm{P}(C = c|Z = z)$ be the sex dependent participation probability. Similarly, let $\pi_{c|gz} = \mathrm{P}(C = c|G = g, Z = z)$ be the sex dependent participation probability conditioned on genotype. Then, the following lemma holds

Lemma 1 (Nguyen et al. 2019)**.**

$$\exp\widetilde{\beta}_{g\to z} = \frac{\pi_{1|(g+1)1}\pi_{1|g0}}{\pi_{1|g1}\pi_{1|(g+1)0}}$$

Proof of Lemma 1.

Proof. By definition, the effect size under the logit model can be represented as an odds ratio such that

$$\exp\widetilde{\beta}_{g\to z} = \frac{\mathrm{P}(Z = 1|G = g+1, C = 1)/\mathrm{P}(Z = 1|G = g, C = 1)}{\mathrm{P}(Z = 0|G = g+1, C = 1)/\mathrm{P}(Z = 0|G = g, C = 1)} \tag{1}$$

which holds for any value of g. Note that we used $g + 1$ to present a genotype change by a single standardized unit.
Since the following equation holds,

$$\begin{aligned}\mathrm{P}(Z = z|G = g, C = c) &= \frac{\mathrm{P}(Z = z, G = g, C = c)}{\mathrm{P}(G = g, C = c)}\\ &= \frac{\mathrm{P}(C = c|G = g, Z = z)\mathrm{P}(G = g, Z = z)}{\mathrm{P}(G = g, C = c)}\\ &= \frac{\pi_{c|gz}\mathrm{P}(G = g, Z = z)}{\mathrm{P}(G = g, C = c)}\end{aligned} \tag{2}$$

we can substitute the elements on the right-hand side of the equation 1 with equation 2. Then, given the independence between G and Z ($P(G = g, Z = z) = P(G = g)P(Z = z)$), all terms other than $\pi_{c|gz}$ cancel out and the equation is simplified to the desired result. □

Based on this lemma, we can obtain the following theorem that shows that the effect of genetic participation ($\beta_{g\to c}$) can be obtained without observing samples of $C = 0$ (samples who did not participate) under the assumption of no sex-gene interaction.

Theorem 1. *Under the logit model described above, the following holds:*

$$\widetilde{\beta}_{g\to z} = -\beta_{g\to c}(\pi_{1|1} - \pi_{1|0})$$

Proof of Theorem 1.

Proof. The proof is based on Nguyen et al. with slight modification [3]. We assume that the effect of variant g on C is small, i.e. $\beta_{g\to c}$ is small. For notational convenience, let us define

$$\begin{aligned} f(x) &= \frac{e^x}{1+e^x} \\ \log f(x) &= x - \log(1+e^x) \\ (\log f(x))' &= \frac{1}{1+e^x} = 1 - f(x) \end{aligned}$$

The participation probability $\pi_{1|gz}$ can be represented as the expected value of participation (because we defined participation as 0 and 1),

$$\begin{aligned} \pi_{1|gz} &= \mathbb{E}[\mathbb{E}[C|G_j = g, Z = z, \epsilon]|G_j = g, Z = z] \\ &= \mathbb{E}[f(\beta_0 + \beta_{g\to c}g + \beta_{z\to c}z + \epsilon)|G_j = g, Z = z] \end{aligned}$$

which has no closed form expression. However, this expectation (or integration, equivalently) can be approximated to the following [4]:

$$\pi_{1|gz} \approx f\left(\frac{\beta_0 + \beta_{g\to c}g + \beta_{z\to c}z}{\sqrt{1+\frac{\pi\sigma^2}{8}}}\right)$$

This approximation holds because the Gauss error function (erf) approximates the logistic function.

Let $\gamma = \sqrt{1+\frac{\pi\sigma^2}{8}}$ and $k_g = \frac{\beta_0+\beta_{z\to c}z}{\gamma}$. Taking the difference between $\log \pi_{1|(g+1)z}$ and $\log \pi_{1|gz}$ gives

$$\begin{aligned} \log \pi_{1|(g+1)z} - \log \pi_{1|gz} &= (1 - f(\xi + k_g)) \cdot \beta_{g\to c}/\gamma \quad \text{(by mean value theorem, there exists } 0 < |\xi| < |\beta_{g\to c}|/\gamma) \\ &\approx (1 - \pi_{1|z}) \cdot \beta_{g\to c}/\gamma \quad (\xi \approx 0 \text{ because } |\xi| < |\beta_{g\to c}|) \end{aligned}$$

Now from **Lemma 1**,

$$\begin{aligned} \widetilde{\beta}_{g\to z} &= \log\left(\frac{\pi_{1|(g+1)1}/\pi_{1|g1}}{\pi_{1|(g+1)0}/\pi_{1|g0}}\right) \\ &= (1-\pi_{1|1})\beta_{g\to c}/\gamma - (1-\pi_{1|0})\beta_{g\to c}/\gamma \\ &= -\frac{\beta_{g\to c}}{\gamma} \cdot (\pi_{1|1} - \pi_{1|0}) \end{aligned}$$

Pirastu et al. assumed a small $\sigma = 0.01$ for simulation in their study [2]. We similarly assumed a small σ in our study. If σ is small, γ approximates to 1, which completes the proof.

□

The theorem above has the following implications. The theorem provides an analytic relationship between the spurious association between sex and autosomal variants, and the contribution of variant g on study participation. The most important implication of the theorem is that although $\beta_{g\to c}$ can not be directly obtained from the data since samples with $C = 0$ are never observed, it can be inferred using only the sampled data by multiplying a constant factor $(\pi_{c|1} - \pi_{c|0})^{-1}$ to $\beta_{g\to z}$ which is possible to estimate.

The second implication is that it provides a formal justification behind the direction of the collider bias given the direction of the correlation between the causes and the collider. Griffith et al. used an example where the admission into prestigious schools is affected by both sporting ability and academic ability [5]. Based on a graphical illustration, they argued that a negative association between sporting ability and academic ability is induced by conditioning on admission, since both factors positively affect admission into prestigious schools. We present the first formal proof for the same assertion under a GWAS context. If Z and G both increase the probability of study participation, i.e. $\pi_{c|1} > \pi_{c|0}$ and $\beta_{g\to c} > 0$, the theorem clearly shows that the direction of the bias will be negative as asserted by Griffith et al. This however, as pointed out by Shahar and Shahar, may not strictly hold because the particular direction of the collider bias can depend on multiple factors other than the sign of the effect of exposures [6].

4 The sex-gene interaction model

Although **Theorem 1** shows a simple relationship between $\beta_{g\to c}$ and $\beta_{g\to z}$, this simple model and relationship do not sufficiently explain the observed data. Note that **Theorem 1** holds for all variants, and thus can be applied to heritability. That is, we can expect the following relationship to hold under this simple model:

$$h_Z^2 = h_C^2(\pi_{1|1} - \pi_{1|0})^2$$

In the UK Biobank data, h_Z^2 was approximately 2.3%. However, the estimate of between-sex study participation difference $\pi_{1|1} - \pi_{1|0}$ in the UK Biobank was around 1.3% [7, 8]. Therefore, even if h_C^2 has its maximum value of 1.0 (because heritability is 1 at its maximum), the equation can not hold.

Pirastu et al. reported a similar conclusion that the simple model without interaction cannot explain the observed data, although their argument was based on simulations rather than analytical formulation. Based on extensive simulations, they showed that if the effect of a heritable trait on study participation depends on sex (sex-gene interaction), then the collider bias can explain the observed heritability of sex in the UK Biobank [2]. In other words, by simulations, they found that a sex-gene interaction is a core component that can explain the observed association between autosomal variants and sex.

To incorporate sex-gene interaction, we add an interaction term to our previous model.

$$\log\left(\frac{\pi}{1-\pi}\right) = \beta_0 + \beta_{g\to c}G_j + \beta_{z\to c}Z + \beta_{zg\to c}ZG_j + \epsilon$$

The following theorem provides a theoretical and analytical foundation of what Piratsu et al. found by simulations.

Theorem 2. *Under the sex-gene interaction model, the following holds:*

$$\widetilde{\beta}_{g\to z} = -\beta_{g\to c}(\pi_{1|1} - \pi_{1|0}) + \beta_{zg\to c}(1 - \pi_{1|1})$$

Proof of Theorem 2.

Proof. The proof is nearly identical to the proof of **Theorem 1**. The difference comes from the following modification to $\pi_{1|gz}$ by adding a sex-gene interaction term.

$$\begin{aligned}\pi_{1|gz} &= \mathbb{E}[\mathbb{E}[C|G_j = g, Z = z, \epsilon]|G_j = g, Z = z] \\ &= \mathbb{E}[f(\beta_0 + \beta_{g\to c}g + \beta_{z\to c}z + \beta_{zg\to c}zg + \epsilon)|G_j = g, Z = z]\end{aligned}$$

Then by a similar argument we have the following:

$$\begin{aligned}&\log \pi_{1|(g+1)z} - \log \pi_{1|gz} \\ &= (1 - f(\xi + k_g)) \cdot (\beta_{g\to c} + \beta_{zg\to c}z)/\gamma \quad \text{(by mean value theorem)} \\ &\approx (1 - \pi_{1|z}) \cdot (\beta_{g\to c} + \beta_{zg\to c}z)/\gamma \quad (\beta_{g\to c} + \beta_{zg\to c}z \text{ is small and } 0 < |\xi| < |\beta_{g\to c} + \beta_{zg\to c}z|/\gamma)\end{aligned}$$

. From **Lemma 1**,

$$\begin{aligned}\widetilde{\beta}_{g\to z} &= \log\left(\frac{\pi_{1|(g+1)1}/\pi_{1|g1}}{\pi_{1|(g+1)0}/\pi_{1|g0}}\right) \\ &= (1 - \pi_{1|1})(\beta_{g\to c} + \beta_{zg\to c})/\gamma - (1 - \pi_{1|0})\beta_{g\to c}/\gamma \\ &= -\frac{\beta_{g\to c}}{\gamma} \cdot (\pi_{1|1} - \pi_{1|0}) + \frac{\beta_{zg\to c}}{\gamma} \cdot (1 - \pi_{1|1})\end{aligned}$$

if σ^2 is small, γ approximates to 1, which completes the proof.

□

The theorem has the following interpretation. As the current participation rate of genomic cohorts (such as the UKB) respect to the total population is fairly around 5%, assuming that $\beta_{g\to c}$ and $\beta_{zg\to c}$ has similar orders of magnitude, **Theorem 2** shows that the latter term $\beta_{zg\to c}(1-\pi_{1|1})$ is the dominant factor driving the association between autosomal variants and sex. Thus, it is not surprising that Piratsu et al. were able to explain the observed sex-gene association by the simulations incorporating the interaction.

This theorem not only explains the observation of Pirastu et al. but also allows several important predictions. As the sample size grows, the former term $\beta_{g\to c}(\pi_{1|1}-\pi_{1|0})$ can become important in future studies. This is because of the following reason. The increased participation π can be interpreted as the increase in the intercept β_0, given that all other β were unchanged. However, since this is a logistic model, the difference $\pi_{1|1}-\pi_{1|0}$ will gradually increase as the participation rate increases to around 0.5 (due to the slope change in the logistic curve). Thus, the first term will become larger. Conversely, the second term that is expected to drive sex-autosome association at the present state will become less important as $1-\pi_{1|1}$ becomes smaller. Consequently, the nature of collider bias will change gradually as the sample size grows in the future.

The formula can also be used to predict genetic correlation of sex and other traits. From **Theorem** 2, we have

$$\begin{aligned} cov(\beta_{g\to y}, \widetilde{\beta}_{g\to z}) &= -cov(\beta_{g\to y}, \beta_{g\to c}(\pi_{1|1}-\pi_{1|0})) + cov(\beta_{g\to y}, \beta_{zg\to c}(1-\pi_{1|1})) \\ &= -(\pi_{1|1}-\pi_{1|0})cov(\beta_{g\to y}, \beta_{g\to c}) + (1-\pi_{1|1})cov(\beta_{g\to y}, \beta_{zg\to c}) \end{aligned}$$

where the two correlation terms correspond to the genetic correlation of Y and C for the main term and the interaction term respectively. However, the two correlations cannot be recovered from the data because we cannot obtain the summary statistics of C as $C=0$ is not observed.

For completeness, we state the following and end this section.

Theorem 3. *Model A, B and C of Pirastu et al. are all special cases of the sex-gene interaction model.*

Proof of Theorem 3.

Proof. Simplifying the logit model (without interaction) in Section 3 by setting the intercept β_0 to zero leads us to Model A and Model B. Given the logit model with interaction in Section 4, setting the intercept to zero and setting $\beta_{zg\to c}$ to $(K-1)\beta_{g\to c}$ leads us to Model C. (Recall that, we defined K as a constant depicting modifying effect of Z on Y in Section 2.) □

Combining **Theorem 1**, **2** and **3** proves why Model C could explain the observed autosomal heritability of sex under realistic parameters but Model A and B could not. The following section discusses the effect of sex-independent and differential participation on the genotype-trait associations of other traits rather than sex.

5 The effect of sex-differential participation on the genotype-trait associations of other traits

We first introduce notations. Let Y be the trait of interest, which has causal effect on participation. $\beta_{g\to c}^{Y=y,Z=z}$ is the effect of variant j on participation conditioned on phenotype $Y = y$ and sex $Z = z$. $\pi_{c|gyz} = P(C = c|G_j = g, Y = y, Z = z)$ is the participation probability conditioned on $G_j = g$, $Y = y$, $Z = z$. Occasionally, we will omit the first subscript in $\pi_{c|gyz}$ and write $\pi_{c|yz}$ since the effect of a genetic variant is negligible according to the infinitesimal model. Then we can assume a logit model with sex-specific effects of G_j and Y on C.

$$\log\left(\frac{\pi}{1-\pi}\right) = \beta_0 + \beta_{g\to c}^{Y=y,Z=z} G_j + \beta_{y\to c}^{Z=z} Y + \beta_{z\to c} Z + \epsilon$$

Based on this notation, the following DAG depicts the sex-differential bias.

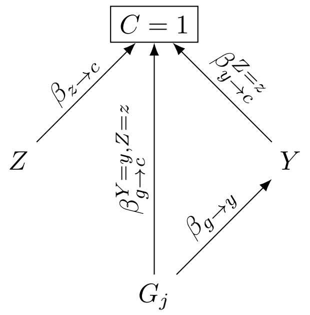

Under this model, the collider bias has an analytic expression:

Theorem 4. *Under sex-differential participation, the log odds ratio of G_j on Y conditional on $C = 1$ is*

$$\widetilde{\beta}_{g\to y} = \beta_{g\to y} + \sum_z \left[\frac{\pi_{1|1z}(1-\pi_{1|1z})}{\sum_{z'} \pi_{1|1z'}} \beta_{g\to c}^{Y=1,Z=z} - \frac{\pi_{1|0z}(1-\pi_{1|0z})}{\sum_{z'} \pi_{1|0z'}} \beta_{g\to c}^{Y=0,Z=z} \right]$$

Theorem 4 follows from the following two lemmas:

Lemma 2 (Nguyen et al. 2019). *Let $\beta_{g\to y}$ be the log odds ratio of variant j in the absence of selection bias. Then the log odds ratio under selection is*

$$\widetilde{\beta}_{g\to y} = \beta_{g\to y} + \log \frac{\pi_{1|(g+1)1}\pi_{1|g0}}{\pi_{1|(g+1)0}\pi_{1|g1}}$$

Lemma 3 (Multivariate mean-value theorem)**.** *Let A be open in $\mathbb{R}^n$. Suppose that partial derivatives $\frac{\partial f}{\partial x_i}$ exist for $f : A \to R$ and are continuous on A. Then there exist $q_i \in (a_i, h_i)$ for $a, h \in A$ where the following holds*

$$f(a+h) - f(a) = \sum_j \frac{\partial f}{\partial x_i}(q_i)(h_i - a_i)$$

Proof of Lemma 2.

Proof. We start from the last line of the proof of **Lemma 1** (that is, the final form of equation (2)). Now, Y can be any phenotype and not necessarily biological sex. Thus, we can replace Z with Y, as follows.

$$\mathrm{P}(Y = y|G = g, C = c) = \frac{\pi_{c|gy}\mathrm{P}(G = g, Y = y)}{\mathrm{P}(G = g, C = c)}$$

Furthermore,

$$\frac{\pi_{c|gy}\mathrm{P}(G=g,Y=y)}{\mathrm{P}(G=g,C=c)} = \frac{\pi_{c|gy}\mathrm{P}(Y=y|G=g)}{\mathrm{P}(C=c|G=g)}$$

Substituting this formula into left-hand side of the equation (1) gives:

$$\exp\widetilde{\beta}_{g\to y} = \frac{\pi_{1|(g+1)1}\pi_{1|g0}}{\pi_{1|(g+1)0}\pi_{1|g1}} \cdot \frac{\mathrm{P}(Y=1|G=g+1)/\mathrm{P}(Y=1|G=g)}{\mathrm{P}(Y=0|G=g+1)/\mathrm{P}(Y=0|G=g)}$$

The second term in the above product is $\exp\beta_{g\to y}$ according to the definition of the odds ratio.

□

While **Lemma 1** showed the analytic form of the bias when the trait of interest was sex, for which we know there is no association to G, **Lemma 2** shows the analytic form of the bias for any trait that may have association to G. As we can see, the bias form is similar to **Lemma 1** but is presented as an additional term to the original association that would be observed under no selection. We note that the proofs of **Lemma 1** and **Lemma 2** were adopted from Nguyen et al. with some simplifications. We refer to the reference for a more detailed proof [3].

Proof of Lemma 3.

Proof. The proof of the theorem can be found in **Theorem 6.2.** of [9]. □

Proof of Theorem 4.

Proof.

$$\begin{aligned}
\mathrm{P}(C=1|G_j=g,Y=y) &= \sum_z \mathrm{P}(C=1|G_j=g,Y=y,Z=z)\cdot\mathrm{P}(Z=z|G_j=g,Y=y) \\
&= \sum_z \mathrm{P}(C=1|G_j=g,Y=y,Z=z)\cdot\mathrm{P}(Z=z) \\
&= \frac{1}{2}\sum_z \mathrm{P}(C=1|G_j=g,Y=y,Z=z) \\
&\approx \frac{1}{2}\sum_z f\left(\frac{\beta_0+\beta_{g\to c}^{Y=y,Z=z}G_j+\beta_{y\to c}^{Z=z}Y+\beta_{z\to c}Z}{\gamma}\right)
\end{aligned}$$

Let $\delta^{Y=y,Z=z}(g) = \beta_{g\to c}^{Y=y,Z=z}g$, then $\pi_{1|gy}$ can be treated as a two component function $\pi_{1|gy}:\mathbb{R}^2\to\mathbb{R}$ respect to $(\delta^{Y=y,Z=0},\delta^{Y=y,Z=1})$. Applying two-variable mean-value theorem (**Lemma 3**) gives the following approximation:

$$\begin{aligned}
\log\pi_{1|(g+1)y}-\log\pi_{1|gy} &\approx \sum_z \frac{\partial\log\pi_{1|gy}}{\partial\delta^{Y=y,Z=z}}(\delta^{Y=y,Z=z}(g+1)-\delta^{Y=y,Z=z}(g)) \\
&= \sum_z \frac{\partial\log\pi_{1|gy}}{\partial\delta^{Y=y,Z=z}}(\beta_{g\to c}^{Y=y,Z=z}(g+1)-\beta_{g\to c}^{Y=y,Z=z}g) \\
&= \sum_z \frac{\partial\log\pi_{1|gy}}{\partial\delta^{Y=y,Z=z}}\beta_{g\to c}^{Y=y,Z=z}
\end{aligned}$$

The partial derivative $\frac{\partial\log\pi_{1|gy}}{\partial\delta^{Y=y,Z=z}}$ can be calculated using the chain rule:

$$\frac{\partial \log \pi_{1|gy}}{\partial \delta^{Y=y,Z=z}} = \frac{f'\left(\frac{\beta_0+\delta^{Y=y,Z=z}(g)+\beta^{Z=z}_{y\to c}y+\beta_{z\to c}z}{\gamma}\right)}{\sum_{z'} f\left(\frac{\beta_0+\delta^{Y=y,Z=z'}(g)+\beta^{Z=z'}_{y\to c}y+\beta_{z\to c}z'}{\gamma}\right)} \cdot \frac{1}{\gamma}$$

Since $(f'(x) = f(x)(1-f(x)))$,

$$= \frac{1}{\gamma} \cdot \frac{f\left(\frac{\beta_0+\delta^{Y=y,Z=z}(g)+\beta^{Z=z}_{y\to c}y+\beta_{z\to c}z}{\gamma}\right)\left(1-f\left(\frac{\beta_0+\delta^{Y=y,Z=z}(g)+\beta^{Z=z}_{y\to c}y+\beta_{z\to c}z}{\gamma}\right)\right)}{\sum_{z'} f\left(\frac{\beta_0+\delta^{Y=y,Z=z'}(g)+\beta^{Z=z'}_{y\to c}y+\beta_{z\to c}z'}{\gamma}\right)}$$

$$= \frac{1}{\gamma} \cdot \frac{\pi_{1|yz}(1-\pi_{1|yz})}{\sum_{z'} \pi_{1|yz'}}$$

Substituting this result to the second term of **Lemma 2** finishes the proof.

□

Theorem 4 can be further refined to several simpler forms if we impose additional assumptions. We first present the results for the models suggested in Pirastu et al. [2]. In model A, the bias is the same to the sex case (**Theorem 1**) as Z has no role in the collider bias between G_j and Y as shown in the following figure.

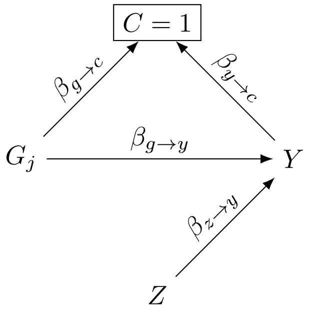

Hence, $\pi_{1|yz} = \pi_{1|y}$ for all $z = 0, 1$. We can obtain the following result directly from **Theorem 1**.

Corollary 1 (Model A)**.**

$$\widetilde{\beta}_{g\to y} = \beta_{g\to y} - \beta_{g\to c}(\pi_{1|1} - \pi_{1|0})$$

Model B and C share the same DAG but $\beta^{Y=y,Z=z}_{g\to c}$ is constant in respect of Y and Z in Model B. This leads to the following result.

Corollary 2 (Model B)**.**

$$\widetilde{\beta}_{g\to y} = \beta_{g\to y} + \beta_{g\to c} \cdot \sum_z \left(\frac{\pi_{1|1z}(1-\pi_{1|1z})}{\sum_{z'} \pi_{1|1z'}} - \frac{\pi_{1|0z}(1-\pi_{1|0z})}{\sum_{z'} \pi_{1|0z'}} \right)$$

In Model C, $\beta^{Y=y,Z=z}_{g\to c}$ is constant only in respect of Y but not Z. The constant K, as in Pirastu et al. [2], is the ratio between $\beta^{Z=1}_{g\to c}$ and $\beta^{Z=0}_{g\to c}$ ($\beta^{Z=1}_{g\to c} = K\beta^{Z=0}_{g\to c}$).

Corollary 3 (Model C)**.**

$$\widetilde{\beta}_{g\to y} = \beta_{g\to y} + \sum_z \left[\frac{\pi_{1|1z}(1-\pi_{1|1z})}{\sum_{z'} \pi_{1|1z'}} - \frac{\pi_{1|0z}(1-\pi_{1|0z})}{\sum_{z'} \pi_{1|0z'}} \right] \beta^{Z=z}_{g\to c}$$

These formulas can be further simplified under the rare outcome assumption. When the participation rate is low, the logit function $\log \frac{x}{1-x}$ is approximately $\log x$. The ratios $\frac{\pi_{1|yz}(1-\pi_{1|yz})}{\sum_{z'} \pi_{1|yz'}}$ can be expressed soley by β variables.

Corollary 4 (Rare participation)**.** *Under low participation rate, Model B simplifies to*

$$\beta_{g\to y} - \beta_{g\to c} \cdot \left[\frac{1}{e^{\beta_{z\to c}}+1}(\pi_{1|10} - \pi_{1|00}) + \frac{e^{\beta_{z\to c}}}{e^{\beta_{z\to c}}+1}(\pi_{1|11} - \pi_{1|01})\right]$$

and Model C simplifies to

$$\beta_{g\to y} + \sum_z \left[\frac{e^{\beta_{y\to c}^{Z=z}+\beta_{z\to c}z}}{e^{\beta_{y\to c}^{Z=0}} + e^{\beta_{y\to c}^{Z=1}+\beta_{g\to z}}}(1-\pi_{1|1z}) - \frac{e^{\beta_{z\to c}z}}{1+e^{\beta_{z\to c}}}(1-\pi_{1|0z})\right]\beta_{g\to c}^{Z=z}$$

Proof. To show this, recall that

$$\pi_{1|ygz} = f\left(\frac{\beta_0 + \beta_{g\to c}^{Y=y,Z=z}g + \beta_{y\to c}^{Z=z}y + \beta_{z\to c}z}{\gamma}\right)$$

Under low participation, β_0 is a substantially large negative value. For example, when participation rate is 5%, β_0 is about $\log(0.05) \approx -3$. Thus, highly likely, the formula inside the parentheses also becomes substantially large negative. We can then approximate the logit function and ignore the effect of G_j

$$\begin{aligned}\pi_{1|ygz} &= f\left(\frac{\beta_0 + \beta_{g\to c}^{Y=y,Z=z}g + \beta_{y\to c}^{Z=z}y + \beta_{z\to c}z}{\gamma}\right)\\ &\approx \exp\left(\frac{\beta_0 + \beta_{g\to c}^{Y=y,Z=z}g + \beta_{y\to c}^{Z=z}y + \beta_{z\to c}z}{\gamma}\right) \quad (f = \tfrac{e^x}{1+e^x} \approx e^x, \text{ if } e^x \text{ is small})\\ &\approx \exp\left(\frac{\beta_0 + \beta_{y\to c}^{Z=z}y + \beta_{z\to c}z}{\gamma}\right) \quad (\text{effect of } G_j \text{ is negligible})\end{aligned}$$

Hence, substituting the result above to $\frac{\pi_{1|yz}}{\sum_{z'}\pi_{1|yz'}}$ and setting $\gamma \approx 1$ (σ is small as in the simulation setting of Pirastu et al.) give the following

$$\begin{aligned}\frac{\pi_{1|yz}}{\sum_{z'}\pi_{1|yz'}} &= \frac{\exp(\beta_0 + \beta_{y\to c}^{Z=z}y + \beta_{z\to c}z)}{\exp(\beta_0 + \beta_{y\to c}^{Z=0}y) + \exp(\beta_0 + \beta_{y\to c}^{Z=1}y + \beta_{z\to c})}\\ &= \frac{\exp(\beta_{y\to c}^{Z=z}y + \beta_{z\to c}z)}{\exp(\beta_{y\to c}^{Z=0}y) + \exp(\beta_{y\to c}^{Z=1}y + \beta_{z\to c})}\end{aligned}$$

and substituting this result into **Theorem 4** gives the desired result. □

6 Sex-stratified analysis can substantially reduce the bias caused by sex-differential participation on the associations of other traits

So far, we have derived the analytical formula for the bias caused by sex-differential participation on the associations of other traits. The formula under low participation rate is particularly of interest, since the current genetic cohorts only recruit a small portion of the target population. Importantly, our formulas can suggest a very simple solution to correct for the bias.

Corollary 4 first shows that under low participation rate, the magnitude of bias can be large if model C is true. It is obvious that $\pi_{1|y1} - \pi_{1|y0}$ is negligible so under Model B, as in the sex GWAS case, the bias is negligible. On the other hand, Model C can cause meaningful shift in the estimates with fairly realistic parameters. To demonstrate this point, we consider a situation in model C with $K = -1$. This means $\beta_{g\to c}^{Z=1} = -1 \cdot \beta_{g\to c}^{Z=0}$ and $\beta_{y\to c}^{Z=1} = -1 \cdot \beta_{y\to c}^{Z=0}$. Then **Corollary 4** is reduced to the following

$$\beta_{g\to y} + \left(\frac{e^{\beta_{y\to c}} - e^{-\beta_{y\to c}+\beta_{z\to c}}}{e^{\beta_{y\to c}} + e^{-\beta_{y\to c}+\beta_{z\to c}}} - \frac{1 - e^{\beta_{z\to c}}}{1 + e^{\beta_{z\to c}}} \right) \beta_{g\to c}$$

Here, we dropped the superscript for sex Z where $\beta_{\bullet}^{Z=0} = \beta_{\bullet}$. We calculated the coefficient of the bias term for a range of values of $\beta_{y\to c}$ and $\beta_{z\to c}$. This is presented in the following table.

OR_Y \ OR_Z	0.37	0.61	1.00	1.65	2.72
0.37	-0.92	-0.88	-0.76	-0.60	-0.44
0.61	-0.46	-0.49	-0.46	-0.39	-0.30
1.00	0.00	0.00	0.00	0.00	0.00
1.65	0.30	0.39	0.46	0.49	0.46
2.72	0.44	0.60	0.76	0.88	0.92

The values indicate that even with modest values of $OR_Y = \exp \beta_{y\to c}$ and $OR_Z = \exp \beta_{z\to c}$, the magnitude of the bias becomes comparable to the true effect size.

This effect is, however, negligible when the GWAS is performed in a sex-stratified manner. The bias term of the sex-stratified analysis can be easily obtained from **Theorem 4**. The rationale is that, for each single-sex analysis (for men and women each), the situation is identical to the case where the likelihood of participation is equal in men and women. Thus, when obtaining the bias for sex $Z = z$, replacing the superscripts of the coefficients of $\beta_{\bullet}^{Z=1-z}$ to $\beta_{\bullet}^{Z=z}$ will reduce the bias term to a very simple form: $-\beta_{g\to c}^{Z=z}(\pi_{1|1z} - \pi_{1|0z})$. Writing this down,

$$\begin{aligned}
\text{bias} &= \sum_z \left[\frac{\pi_{1|1z}(1-\pi_{1|1z})}{\sum_{z'} \pi_{1|1z'}} \beta_{g\to c}^{Y=1,Z=z} - \frac{\pi_{1|0z}(1-\pi_{1|0z})}{\sum_{z'} \pi_{1|0z'}} \beta_{g\to c}^{Y=0,Z=z} \right] \\
&= \sum_z \left[\frac{\pi_{1|1z}(1-\pi_{1|1z})}{\sum_{z'} \pi_{1|1z}} \beta_{g\to c}^{Z=z} - \frac{\pi_{1|0z}(1-\pi_{1|0z})}{\sum_{z'} \pi_{1|0z}} \beta_{g\to c}^{Z=z} \right] \\
&\qquad (z = z' \text{ since only one sex in the analysis}) \\
&= \sum_z \left[\frac{1}{2}(1-\pi_{1|1z})\beta_{g\to c}^{Z=z} - \frac{1}{2}(1-\pi_{1|0z})\beta_{g\to c}^{Z=z} \right] \\
&= \sum_z \left[\frac{1}{2}\beta_{g\to c}^{Z=z}(\pi_{1|0z} - \pi_{1|1z}) \right] \\
&= -\frac{1}{2}\beta_{g\to c}^{Z=0}(\pi_{1|1z} - \pi_{1|0z}) - \frac{1}{2}\beta_{g\to c}^{Z=1}(\pi_{1|1z} - \pi_{1|0z}) \\
&= \begin{cases} -\beta_{g\to c}^{Z=0}(\pi_{1|1z} - \pi_{1|0z}) & \text{if } Z = 0 \\ -\beta_{g\to c}^{Z=1}(\pi_{1|1z} - \pi_{1|0z}) & \text{if } Z = 1 \end{cases} \quad (\text{by switching } \beta_{\bullet}^{Z=1-z} \text{ to } \beta_{\bullet}^{Z=z})
\end{aligned}$$

As this bias is negligible as in **Corollary 1**, single sex analysis is almost immune to the bias from sex-differential participation under the low participation rate situation. Thus, one can substantially reduce the bias by analyzing

each sex separately and meta-analyzing the results, which is a surprisingly simple solution for this complicated problem.

Note that **Theorem 4** is general enough to include the scenario that the effect of variant G_j on participation C is pleiotropic (the effect is mediated by more than one trait). The theorem predicts the bias when running a GWAS on particular trait Y. Then the effect of variant G_j on C that is mediated by Y is depicted in $G_j \rightarrow Y \rightarrow C$ and the rest of the effect that is not mediated by Y (but possibly by traits other than Y) is depicted in $G_j \rightarrow C$. If the effect of G_j on C was totally by Y, only $G_j \rightarrow Y \rightarrow C$ would have been sufficient.

The earlier table demonstrates the degree of bias under the presence of sex imbalance. In our model, sex-imbalance is largely driven by $OR_Z = \exp \beta_{z \rightarrow c}$ which is the odds ratio of a particular sex (e.g. woman) to participate in the study. An interesting observation is that the magnitude of the bias is not simply proportional to the degree of sex imbalance. For example, in the first row of the table where $OR_Y < 1$, the more likely a woman to participate in a study (larger OR_Z), the smaller the bias becomes. On the other hand, in the last row of the table where $OR_Z > 1$, the more likely a woman to participate in a study, larger the bias becomes.

In cases where participation rate is high, we can not use the approximation in **Corollary 4** but it is possible to obtain a similar table of coefficients of the bias term using **Theorem 4**. Again, there is no simple trend in the magnitude of the bias with respect to the degree of sex imbalance. However, the overall magnitude of the bias is much smaller when the participation rate is high, as we can see on the table below where we assumed $\beta_0 = 0$ which corresponds to approximately 50% overall participation.

OR_Y \ OR_Z	0.37	0.61	1.00	1.65	2.72
0.37	-0.14	-0.06	0.00	0.03	0.04
0.61	-0.07	-0.03	0.00	0.02	0.03
1.00	0.00	0.00	0.00	0.00	0.00
1.65	0.04	0.03	0.00	-0.03	-0.04
2.72	0.04	0.03	0.00	-0.05	-0.09

From this table, again, we can see that the degree and the direction of sex imbalance are not sufficient to predict the direction and the magnitude of the bias.

7 Accounting for sex-biased population prevalence

Until now, we have not considered the difference in true trait prevalence between men and women in population. This was because earlier simulation-based studies also have not considered this situation. For example, as shown in the **Chapter 2**, model C does not contain an arrow from Z to Y. Although model A has an arrow from Z to Y, we have shown earlier that sex has no role in the collider bias (**Corollary 1**). Now we consider the most general scenario where we add an arrow from Z to Y to **Theorem 4**.

Theorem 5. *Under sex-differential participation and sex-biased prevalence, the log odds ratio of G_j on Y conditional on $C = 1$ is*

$$\beta_{g\to y} + \sum_z \left[\frac{\phi_{z|1}\pi_{1|1z}(1-\pi_{1|1z})}{\sum_{z'}\phi_{z'|1}\pi_{1|1z'}}\beta_{g\to c}^{Y=1,Z=z} - \frac{\phi_{z|0}\pi_{1|0z}(1-\pi_{1|0z})}{\sum_{z'}\phi_{z'|1}\pi_{1|0z'}}\beta_{g\to c}^{Y=0,Z=z} \right]$$

where $\phi_{z|y} = P(Z=z|Y=y) = \frac{P(Y=y|Z=z)P(Z=z)}{P(Y=y)}$.

Proof of Theorem 5.

Proof. The proof can be done by a very similar manner to previous theorems.

$$\begin{aligned}
\mathrm{P}(C=1|G_j=g, Y=y) &= \sum_z \mathrm{P}(C=1|G_j=g, Y=y, Z=z)\cdot \mathrm{P}(Z=z|G_j=g, Y=y) \\
&\approx \sum_z \mathrm{P}(C=1|G_j=g, Y=y, Z=z)\cdot \mathrm{P}(Z=z|Y=y) \quad \text{(the effect of } G_j \text{ is small)} \\
&\approx \sum_z f\left(\frac{\beta_0 + \beta_{g\to c}^{Y=y,Z=z}G_j + \beta_{y\to c}^{Z=z}Y + \beta_{z\to c}Z}{\gamma}\right)\cdot \phi_{z|y}
\end{aligned}$$

Let $\delta^{Y=y,Z=z}(g) = \beta_{g\to c}^{Y=y,Z=z}g$, then $\pi_{1|gy}$ can be treated as a two component function $\pi_{1|gy}:\mathbb{R}^2 \to \mathbb{R}$ respect to $(\delta^{Y=y,Z=0}, \delta^{Y=y,Z=1})$. Applying two-variable mean-value theorem and the chain rule gives the following approximation:

$$\begin{aligned}
\log \pi_{1|(g+1)y} - \log \pi_{1|gy} &\approx \sum_z \frac{\partial \log \pi_{1|gy}}{\partial \delta^{Y=y,Z=z}}(\delta^{Y=y,Z=z}(g+1) - \delta^{Y=y,Z=z}(g)) \\
&= \sum_z \frac{\partial \log \pi_{1|gy}}{\partial \delta^{Y=y,Z=z}}(\beta_{g\to c}^{Y=y,Z=z}(g+1) - \beta_{g\to c}^{Y=y,Z=z}g) \\
&= \sum_z \frac{\partial \log \pi_{1|gy}}{\partial \delta^{Y=y,Z=z}}\beta_{g\to c}^{Y=y,Z=z}
\end{aligned}$$

Substituting this result to **Lemma 2** finishes the proof. The calculation of the partial derivative $\frac{\partial \log \pi_{1|gy}}{\partial \delta^{Y=y,Z=z}}$ is identical to **Theorem** 4.

□

Both the theorem and the proof are nearly the same as **Theorem 4**. The prevalence of Y depending on the sex, namely $P(Y=y|Z=z)$ enters the equation through the Bayes rule $\phi_{z|y} = P(Z=z|Y=y) = \frac{P(Y=y|Z=z)P(Z)}{P(Y)}$.

8 Appendix: Methods

8.1 Trait simulation

Trait was simulated using Hail v0.2.74. Genotype was generated using the `balding_nicholas_model` function. The `ldscsim` function can simulate trait with differing heritability. The generated trait were standardized to mean 0 and variance 1 by default. Binary traits were generated by treating the continuous trait simulated by `ldscsim` as the underlying liability. Sex was assigned randomly to individuals with probability 0.5. Specific simulation parameters are listed in the corresponding subsections. Study participation was simulated using the same model in **Section 4**. Total 50,000 individuals and 1,000 markers were simulated by the `balding_nicholas_model` function .

8.2 Sex GWAS χ^2 versus participation rate experiment

For sex-differential participation, the following pairs of parameters were used.

**Male** h^2	**Female** h^2	**Between-sex** r_g
0.2	0.05	1
0.4	0.1	1
0.05	0.05	-1

For sex-independent selection, heritability was set to $h^2 = 1$ and effect of sex was set to $\beta_{z\to c} = \log(2) \approx 0.6993$. Participation rate was controlled by changing β_0 from -3 to 3. In the supplementary figures, the simulations were done with $h_C^2 \in \{0, 0.1, 0.2, 0.3\}$, $P(Y = 1) \in \{0.1, 0.5\}$, $r_g \in \{-1, 0, 1\}$ and $P(Y = 1|Z = z) = 0.1 + 0.2z$.

8.3 Robustness of sex-stratified GWAS

Study participation C was generated to have heritability $h^2 = 0.2$ in both sex. The odds ratio of trait y on study participation was set to $|\beta_{y\to c}^{Z=z}| = 0.5$. The sign of the parameter was 1 in female and 0 in male. Trait y was generated with $h^2 = 0$. Participation rate was controlled by changing β_0 from -3 to 3. GWAS was performed using Hail with the combined simulated data and the sex-stratified simulated data. Fixed-effects meta-analysis estimates were produced by combining male and female estimates by inverse variance weighting. In the supplementary figures, the simulations were done with $\beta_0 \in \{-4, -3, -2, \dots, 4\}$, $|\beta_{y\to c}^{Z=z}| = 0.5$, $h_C^2 \in \{0, 0.1, 0.2, 0.3\}$ and $r_g \in \{-1, 0, 1\}$.

8.4 Bias in MR-IVW versus study participation rate experiment

Study participation C was generated to have heritability $h^2 = 0.3$ in both sex. Binary trait y_1 and y_2 were generated to have heritability $h^2 = 0$. Participation rate was controlled by changing β_0 from -3 to 3. GWAS was performed for y_1 and y_2 with the combined simulated data and the sex-stratified simulated data.

MR-IVW estimates were obtained using the summary statistics generated from the previous step. The IVW method used in the study was adopted from Bowden et al. [10].

8.5 β difference between sex and sex GWAS

The following parameter configuration was used.

**Male** h^2	**Female** h^2	**Between-sex** r_g
0.1	0.1	-1

The x-axis was the difference of $\beta_{g\to c}^{Z=z}$ for $z = 0, 1$ and the y-axis was the result of sex GWAS performed using Hail.

References

[1] J. Pearl, *Causality*. Cambridge, UK: Cambridge University Press, 2 ed., 2009.

[2] N. Pirastu, , M. Cordioli, P. Nandakumar, G. Mignogna, A. Abdellaoui, B. Hollis, M. Kanai, V. M. Rajagopal, P. D. B. Parolo, N. Baya, C. E. Carey, J. Karjalainen, T. D. Als, M. D. V. der Zee, F. R. Day, K. K. Ong, T. Morisaki, E. de Geus, R. Bellocco, Y. Okada, A. D. Børglum, P. Joshi, A. Auton, D. Hinds, B. M. Neale, R. K. Walters, M. G. Nivard, J. R. B. Perry, A. Ganna, and and, "Genetic analyses identify widespread sex-differential participation bias," *Nature Genetics*, vol. 53, pp. 663–671, Apr. 2021.

[3] T. Q. Nguyen, A. Dafoe, and E. L. Ogburn, "The magnitude and direction of collider bias for binary variables," *Epidemiologic Methods*, vol. 8, Mar. 2019.

[4] G. E. Crooks, "Logistic approximation to the logistic-normal integral," 2013.

[5] G. J. Griffith, T. T. Morris, M. J. Tudball, A. Herbert, G. Mancano, L. Pike, G. C. Sharp, J. Sterne, T. M. Palmer, G. D. Smith, K. Tilling, L. Zuccolo, N. M. Davies, and G. Hemani, "Collider bias undermines our understanding of COVID-19 disease risk and severity," *Nature Communications*, vol. 11, Nov. 2020.

[6] D. J. Shahar and E. Shahar, "A theorem at the core of colliding bias," *The International Journal of Biostatistics*, vol. 13, Jan. 2017.

[7] N. M. Davies, M. Dickson, G. D. Smith, G. J. van den Berg, and F. Windmeijer, "The causal effects of education on health outcomes in the UK biobank," *Nature Human Behaviour*, vol. 2, pp. 117–125, Jan. 2018.

[8] A. Fry, T. J. Littlejohns, C. Sudlow, N. Doherty, L. Adamska, T. Sprosen, R. Collins, and N. E. Allen, "Comparison of sociodemographic and health-related characteristics of UK biobank participants with those of the general population," *American Journal of Epidemiology*, vol. 186, pp. 1026–1034, June 2017.

[9] J. Munkres, *Analysis On Manifolds*. Basic Books, 1991.

[10] J. Bowden, F. D. G. M, C. Minelli, Q. Zhao, D. A. Lawlor, N. A. Sheehan, J. Thompson, and G. D. Smith, "Improving the accuracy of two-sample summary-data mendelian randomization: moving beyond the NOME assumption," *International Journal of Epidemiology*, vol. 48, pp. 728–742, Dec. 2018.

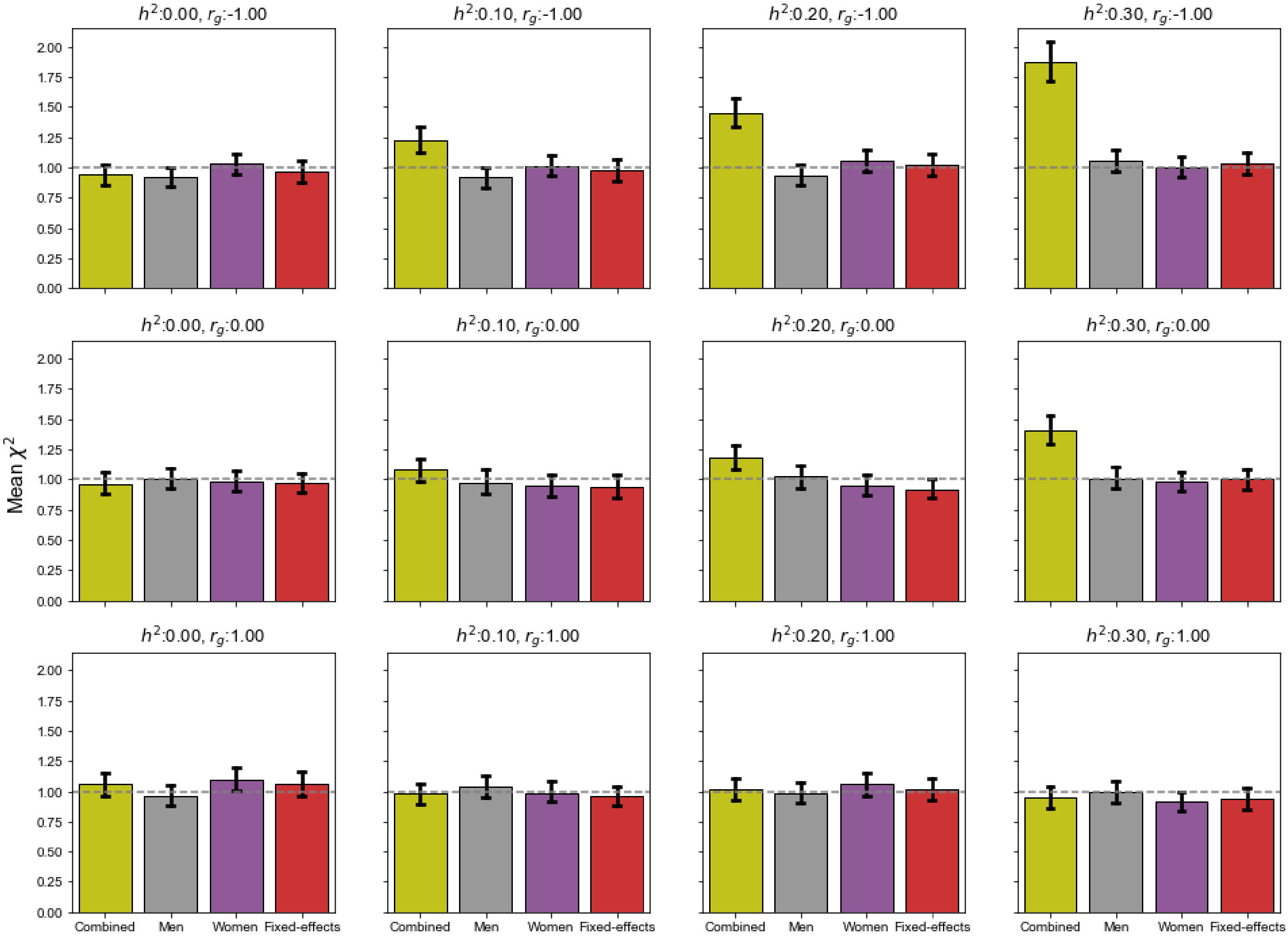

Figure S1. Mean χ^2 statistics of 1,000 variants under sex-differential participation when $P(Y = 1) = 0.5$**.** The compared methods are sex-combined (blue), male-only (orange), female-only (green) and fixed-effects meta-analysis (red). Participation probability was set to $\beta_0 = -4$**.**

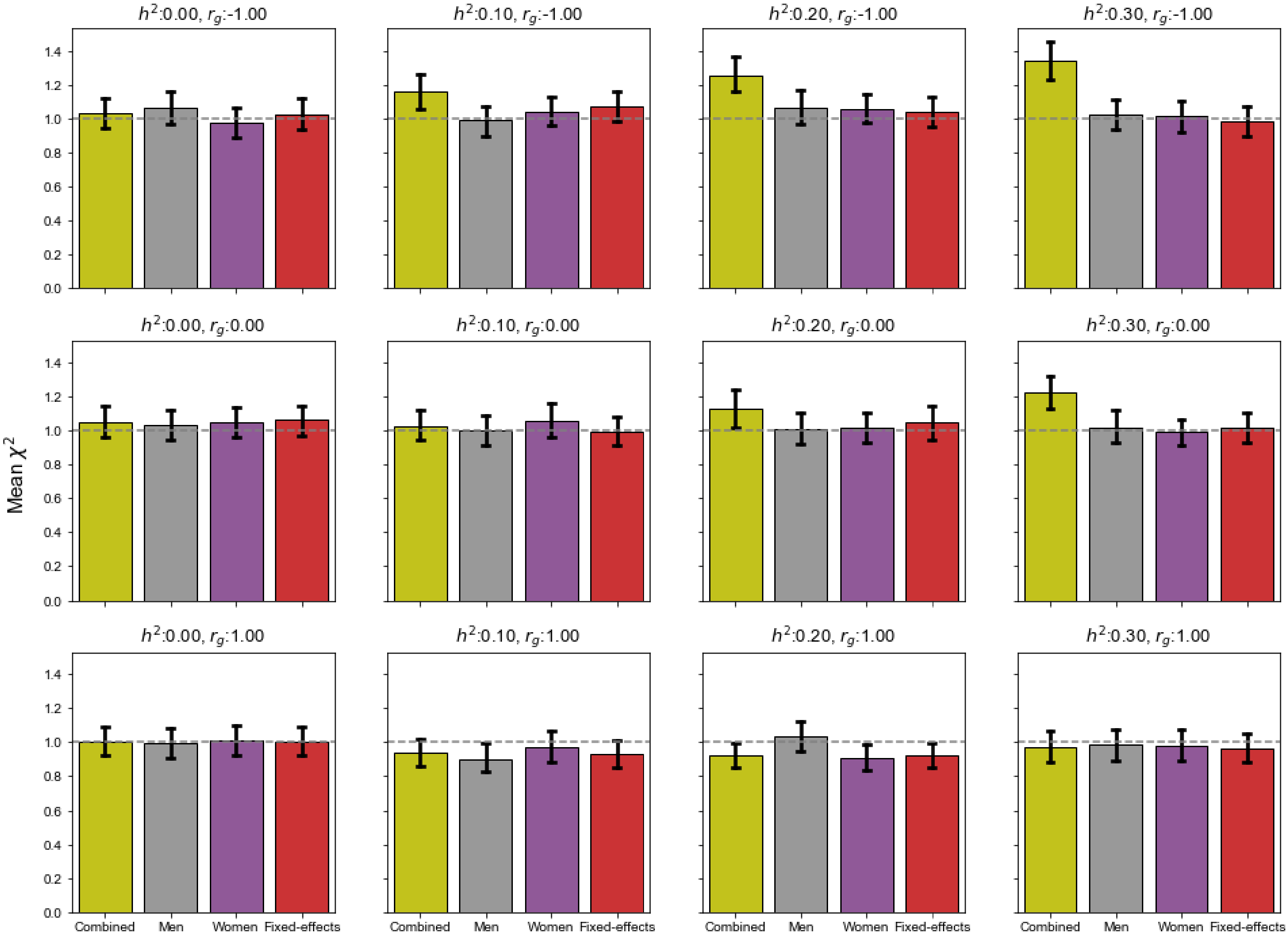

Figure S2. Mean χ^2 statistics of 1,000 variants under sex-differential participation bias $P(Y = 1) = 0.1$**.** The compared methods are sex-combined (blue), male-only (orange), female-only (green) and fixed-effects meta-analysis (red). Participation probability was set to $\beta_0 = -4$**.**

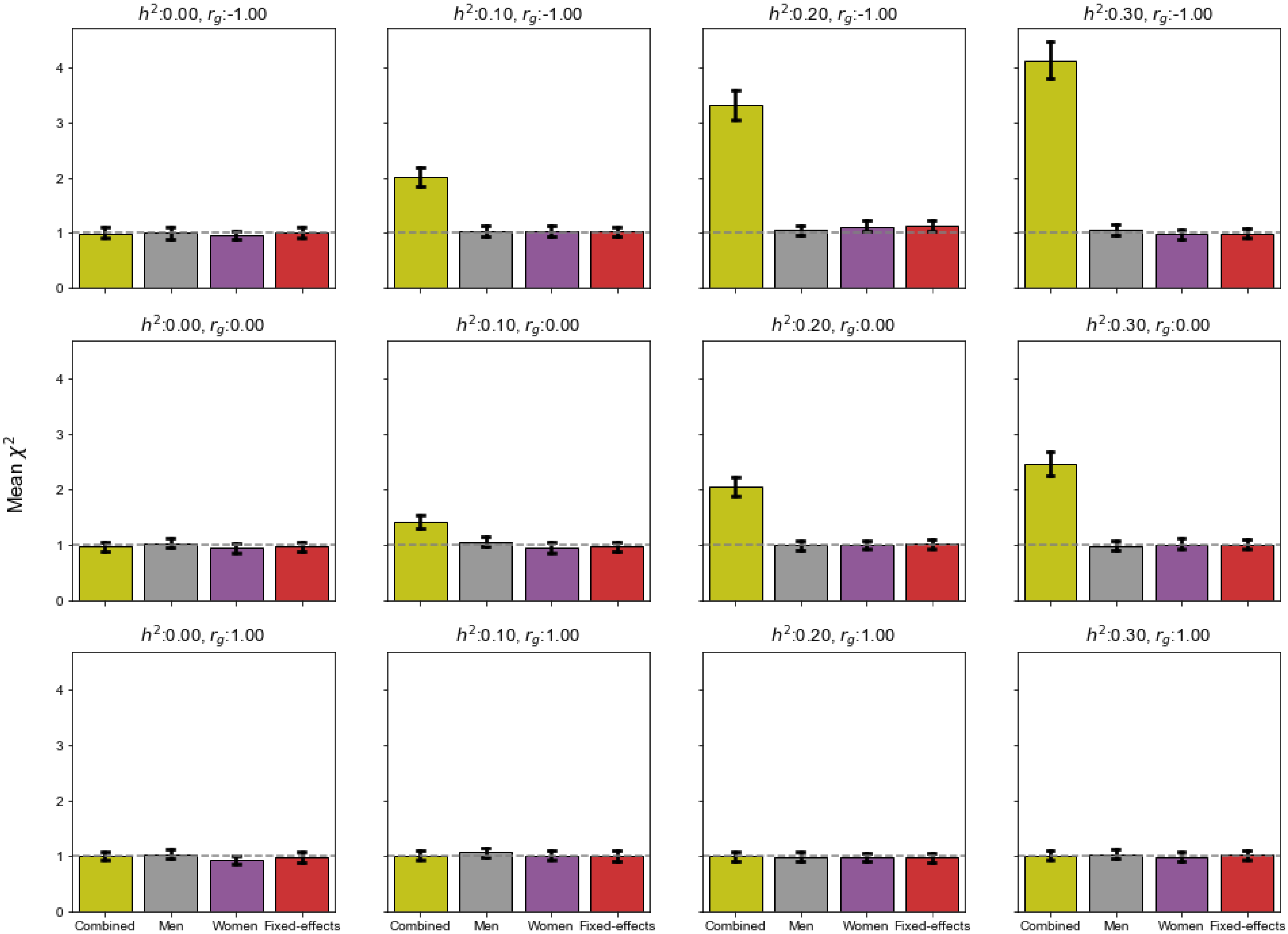

Figure S3. Mean χ^2 statistics of 1,000 variants under sex-differential participation when $P(Y = 1|Z = z) = 0.1 + 0.2z$**.** The compared methods are sex-combined (blue), male-only (orange), female-only (green) and fixed-effects meta-analysis (red). Participation probability was set to $\beta_0 = -4$**.**

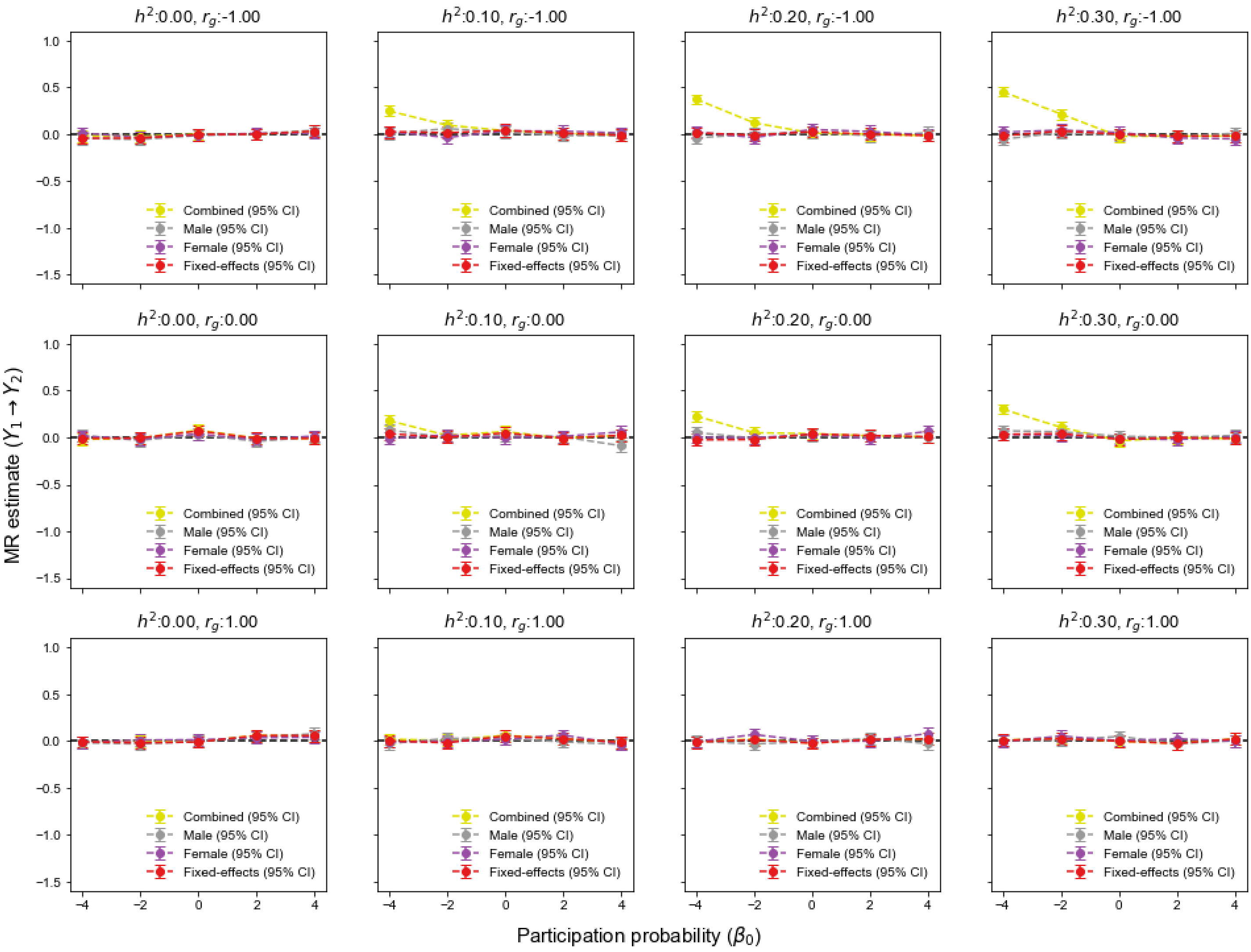

Figure S4. Mendelian randomization estimates of two independent binary traits Y_1 and Y_2 under sex-differential participation when $P(Y = 1) = 0.5$.

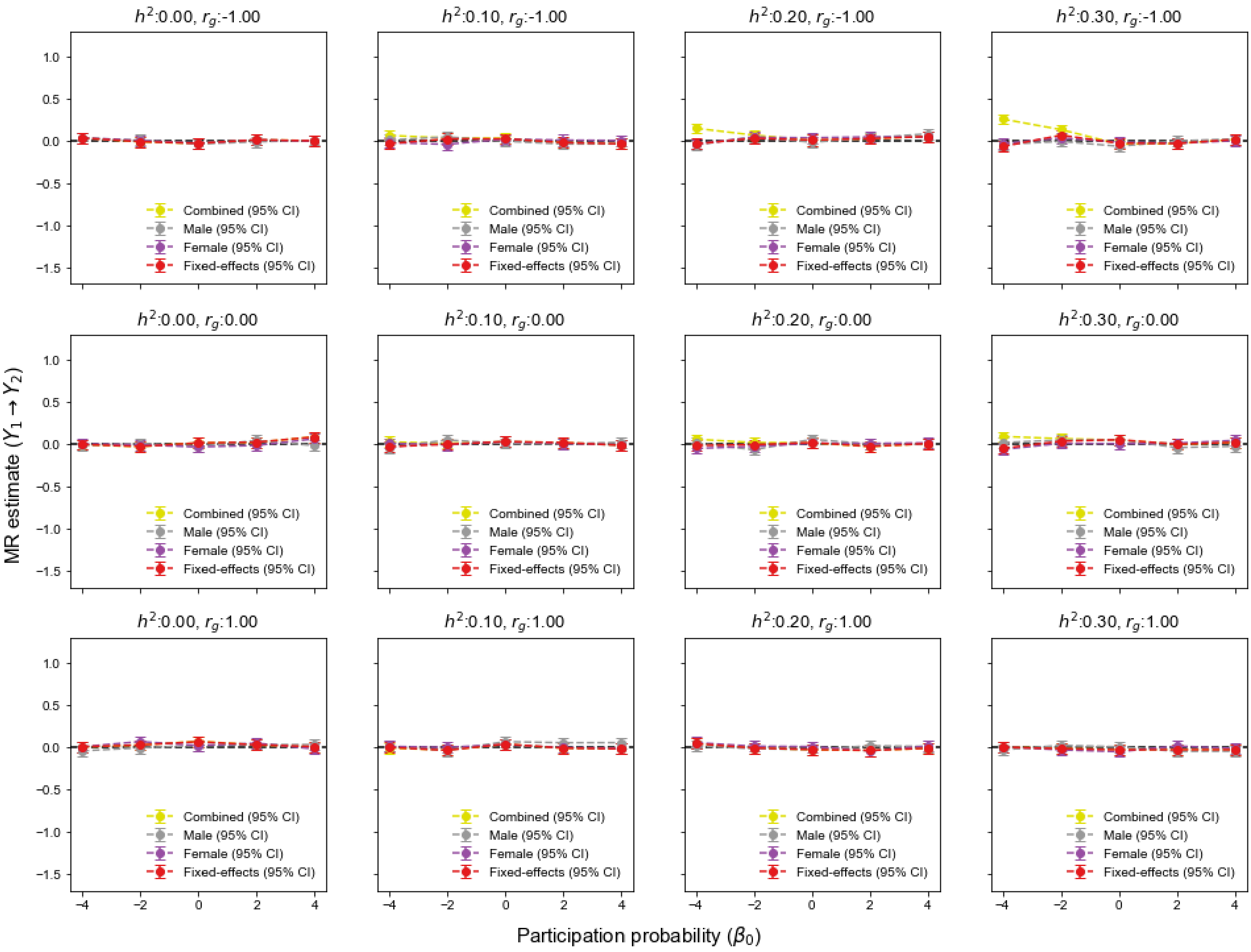

Figure S5. Mendelian randomization estimates of two independent binary traits Y_1 and Y_2 under sex-differential participation when $P(Y = 1) = 0.1$.

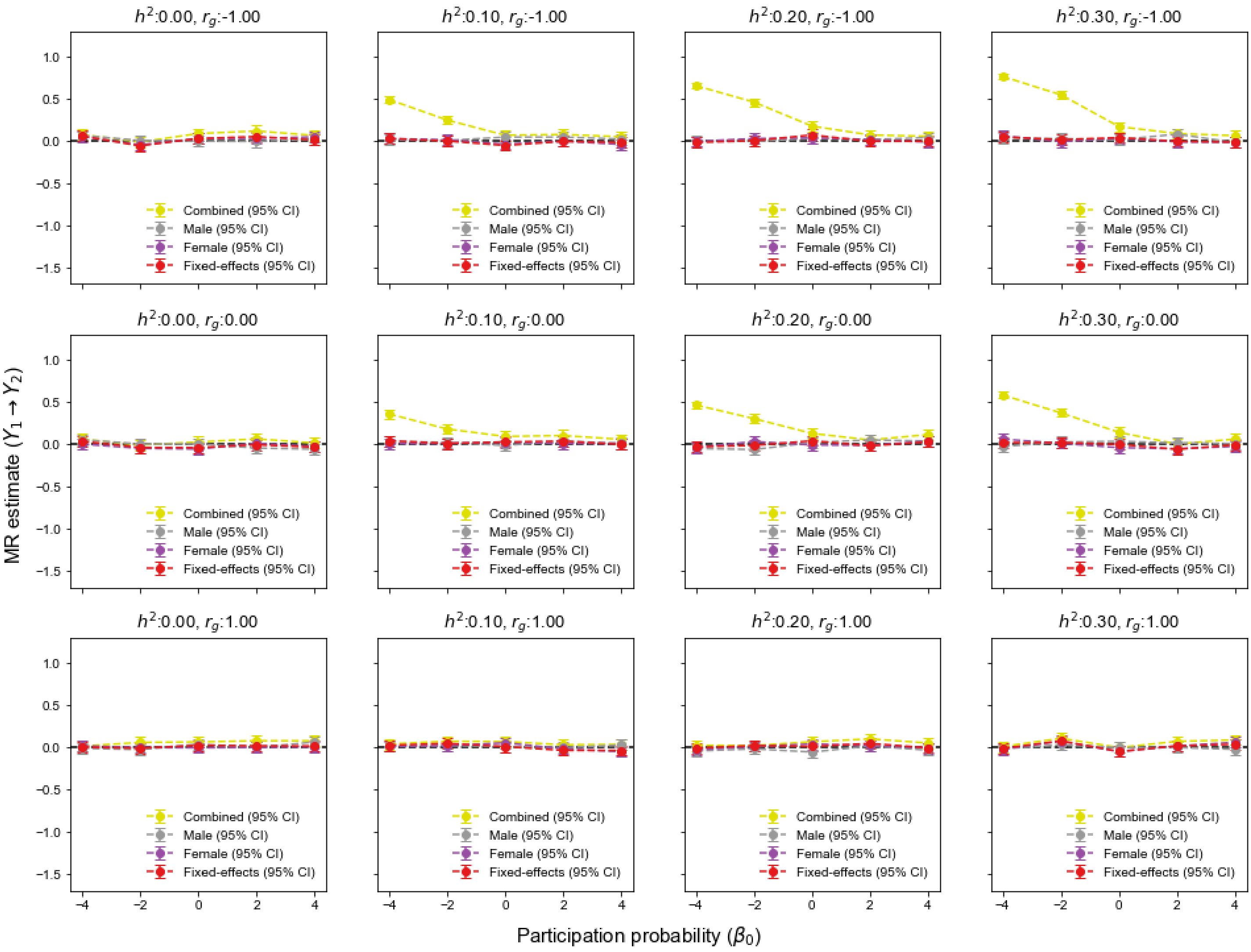

Figure S6. Mendelian randomization estimates of two independent binary traits Y_1 and Y_2 under sex-differential participation when $P(Y = 1|Z = z) = 0.1 + 0.2z$.

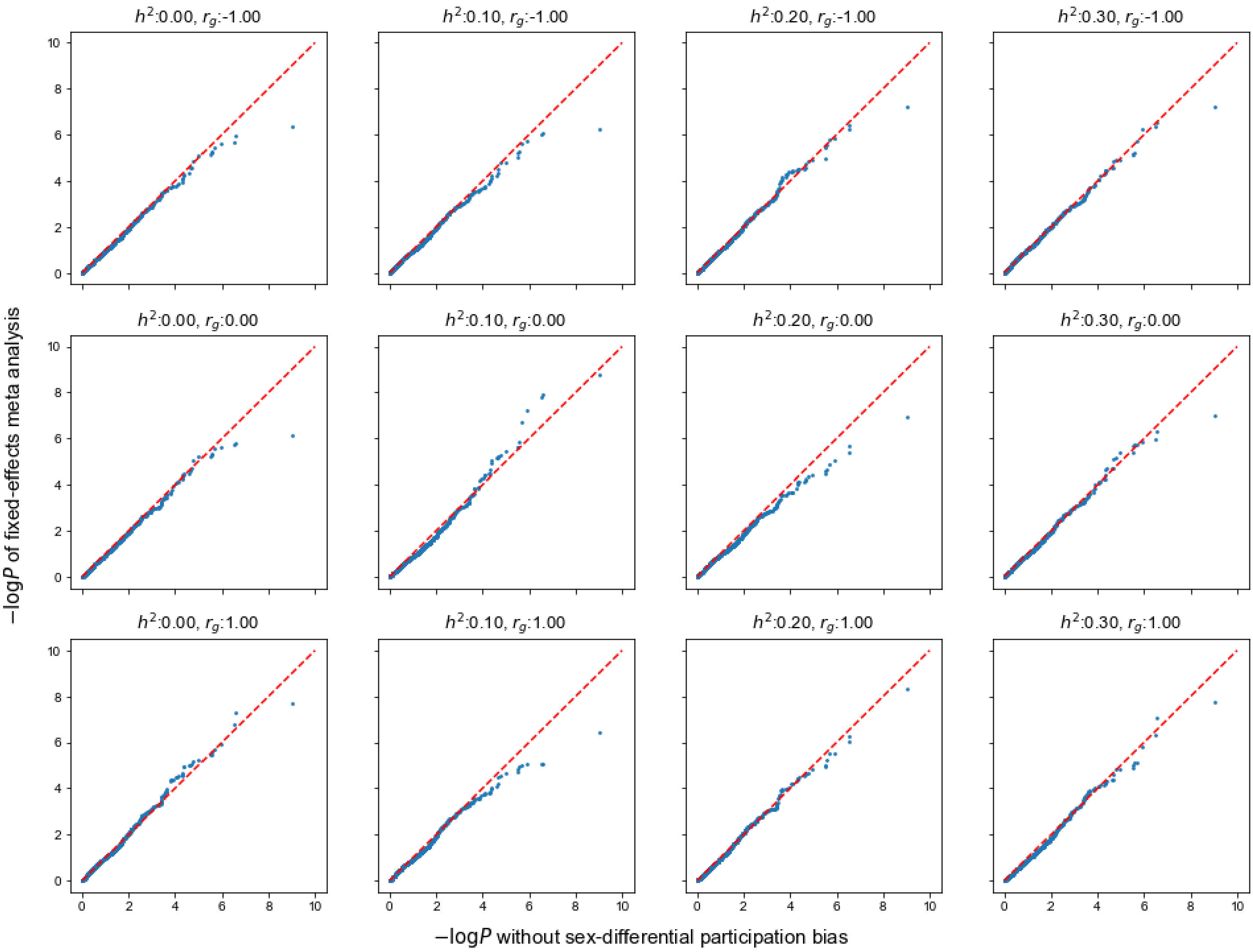

Figure S7. Power of fixed-effects meta-analysis of sex-stratified GWAS. 40,000 participants were simulated for each scenario.
