# Supplementary material for: A theory-based practical solution to correct for sex-differential participation bias
Source: Genome Biol. 2022 Jun 27;23:138. doi: 10.1186/s13059-022-02703-0 (PMC9238114; doi:10.1186/s13059-022-02703-0)
Supplement: Supplementary file 2 — Additional file 2. The review history. [file 13059_2022_2703_MOESM2_ESM.docx]

**1^st^ round**

**Reviewer 1**

This paper by Lee and Han proposes a simple and effective solution to the problem of sex-differentiated selection bias in GWAS, namely, sex stratified GWAS followed by meta-analysis. Furthermore, the authors illustrate the theory behind their assertions that participation rate is a key factor in the degree of sex-differential participation bias. The paper (and supplementary note) is extremely well written, easy to understand, and important as it proposes a clever and simple solution to a real problem. I have only minor suggestions to improve the report, which is already excellent.

1. Can the authors comment on the potential loss of power that occurs when using this approach? I can image this might be a concern for investigators when trying to decide whether to use this approach and it would be good to address it up front.

2. Could the authors provide an example to accompany this point: "Unclear target population definition can reduce the participation rate even if the number of participants increase due to the expansion of the implicit underlying target population." I'm not sure I understand exactly what they mean by "unclear target population definition".

3. The authors describe an important insight into the relationship between study participation rate and selection bias. However, I struggled to understand whether the authors were only addressing the scenario in which the overall participation rate is low, and how variation in the male/female imbalance given participation further affects the bias. Can the authors help the reader develop intuition for the following scenarios…

a) low participation rate overall - high sex imbalance

b) high participation rate overall - high sex imbalance

c) low participation rate overall - low sex imbalance

d) high participation rate overall - low sex imbalance

4. The participation rate discussed here and in referenced papers generally refers to the volunteer participation rate. Can the authors also comment on how these models hold in the context of an electronic health record/biobank study where the "participation rate" may reflect true differences in diagnosis? Furthermore, in a health care setting it is possible for the participation rate to reflect sex-biases in diagnostic practices as well as differences in health-care seeking behaviors between males and females (e.g., males visit the doctor less frequently and when they do visit they receive more diagnoses). It would be helpful for the authors to provide a comment on whether the underlying reason for the participation bias (which may be related to the disease, or related to other factors) matters at all for their proposed solution.

5. Lastly, I would suggest the authors use "between sex" instead of "intersex" throughout the paper. The term "intersex" has both biological and clinical meaning, so used in this context is a bit confusing.

In the interest of accountability in the review process, I choose to sign my reviews, Lea K. Davis.

**Reviewer 2**

In this short report the authors provide a solution to correct for bias in genome-wide association studies due to sex-differential participation bias. Previous studies have shown the presence of such bias, but did not come with a solution. Here, a very logical and easily implementable solution is presented and tested.

Virtually all GWASs using population-based samples will be affected by participation bias, and sex-bias is likely to be an important bias. It's therefore highly relevant and valuable to address this bias and come to a solution. The proposed solution is no rocket science (as the authors notify themselves); it's simple and easy to implement (which is an important aspect). The paper should therefore be of broad interest to human geneticists and the proposed solution represents a significant advance over previous studies. The methodological details are thoroughly described (in the supplementary material) and code has been provided.

The authors show a range of simulations that show the existence of sex-differential participation bias and supports their proposed solution, They may be able to extend their simulations/tests to traits with different prevalences (and prevalence differences between sexes) and a broader range of h2 (and h2 differences between sexes).

Furthermore, although I realise that the paper was submitted as a short report, the discussion may be broadened by discussing downsides of their solution (i.e. splitting samples can reduce statistical power), and by discussing other benefits of splitting samples (sex-differential loci can be detected). I also miss putting the approach in a broader perspective. Sex is one of the potential biases, but there are many others (e.g. age, SES). It's important to address sex-differential participation bias, but shouldn't the participation bias be addressed more generally, and how? This may be beyond the scope of the paper, but it should nevertheless be mentioned and discussed.

In addition, there is another paper on participation bias (and its determinants) that may be relevant to refer to in the manuscript: https://www.nature.com/articles/s41467-021-21073-y

Small remark:

Figure legends. "a loci" is incorrect, it should be "a locus" or "loci"

**Reviewer 3**

In the manuscript entitled "A practical solution to correct for sex-differential participation bias: A theory- based analysis", Lee and Han used theory and simulation to explain the biases in GWAS and follow-up analyses that use GWAS summary statistics due to sex-differential participation bias (i.e., the genetic effects on participation are sex-dependent). Based on their theory and simulation results, we concluded that if the participation rate is low, an efficient solution to correct participation biases is to perform a sex-stratified GWAS analysis. I have several concerns regarding the theoretical derivations and the simulation setting.

The derivations provided in the supplementary note are not easy to follow. For example, it is unclear what is likely to be the most realistic model in practice, and the authors attempted to use their theory to explain the three models proposed in Pirastu et al. without clearly stating what the models are.

I think the SNP effects on participation should be mediated through multiple phenotypes, and the number of phenotypes is likely to be large. However, I do not seem to find such a model in the authors' derivations. It would be interesting to see how the GWAS results for these intermediate traits would be biased by sex-differential participation. In part 4 of the supplementary note, the authors assumed a model where an SNP had a direct sex-dependent effect on participation and an indirect sex-dependent effect mediated through a phenotype. This model is less general than the model above, where all the SNP effects on participation are mediated through phenotypes.

The authors may need to clarify why the bias in the SNP effect on the phenotype due to sex-differential participation bias is independent of the effect of the phenotype on participation in Theorem 4.

The authors derived the theory for a binary phenotype. However, many phenotypes are quantitative. It would be nice if they could extend the theoretical derivations to quantitative phenotypes.

Is it possible to predict by theory the genetic correlation between sex and a phenotype in the presence of participation bias?

Under rare participation, it's unclear how Theorem 4 was simplified for model C.

The authors may want to elaborate on the derivations on how

Theorem 4 was simplified for the sex-stratified analysis.

The standard errors (SEs) of the estimates shown in Figure 1 are all too large. Since the SEs were observed from simulations, the authors can undoubtedly improve the precisions of the estimates by simulating a large sample and performing a larger number of simulations.

I would add the MR result based on a meta-analysis of sex-stratified GWASs in Figure 1c.

**Authors’ response**

Reviewer #1:

This paper by Lee and Han proposes a simple and effective solution to the problem of sex-differentiated selection bias in GWAS, namely, sex stratified GWAS followed by meta-analysis. Furthermore, the authors illustrate the theory behind their assertions that participation rate is a key factor in the degree of sex-differential participation bias. The paper (and supplementary note) is extremely well written, easy to understand, and important as it proposes a clever and simple solution to a real problem. I have only minor suggestions to improve the report, which is already excellent.

Response: We thank the reviewer for the kind words and constructive comments. In particular, we obtained more generalized results thanks to the third comment by the reviewer.

1. Can the authors comment on the potential loss of power that occurs when using this approach? I can image this might be a concern for investigators when trying to decide whether to use this approach and it would be good to address it up front.

Response: As the reviewer pointed out, statistical power is a very important issue for applied researchers who perform GWAS in practice. In theory, meta-analysis (fixed-effects meta-analysis) is unlikely to result in loss of power. Magi et al. (Genetic Epidemiology, 2010) have shown this with both theory and simulations. The point is that whether their results remain solid under collider bias.

We ran simulation comparing the -log(P-value) from GWAS from an unbiased sample and fixed-effects meta-analysis GWAS on a sample with sex-differential participation bias. As we see in the figure, distribution of P-values is nearly the same. The full figure can be found in **Supplementary Figure 7**.

In a theoretical viewpoint, this is predicted by combining Theorem 4 of our work and the work of Magi et al. Theorem 4 guarantees that the parameter (log odds ratio of the variant) being estimated is nearly the same in both sexes. Magi et al. shows that when the parameter being estimated is identical in both sexes, there is no loss of power. Hence, sex-stratified GWAS followed by fixed-effects meta-analysis is unlikely to suffer much from loss of power.

We added the following sentence in the main article:

We show that this stratified approach does not result in loss of statistical power compared to combined GWAS in a representative sample without participation bias (**Supplementary Figure 7**).

Link for Magi et al.: https://onlinelibrary.wiley.com/doi/10.1002/gepi.20540 

2. Could the authors provide an example to accompany this point: "Unclear target population definition can reduce the participation rate even if the number of participants increase due to the expansion of the implicit underlying target population." I'm not sure I understand exactly what they mean by "unclear target population definition".

Response: We apologize for the unclear description of what we meant to say. The target population is the population in which we investigate. For example, the UK Biobank collected residents of the United Kingdom within 40-69 age group at the time of investigation. On the other hand, 23andMe is a commercial cohort where anyone who are willing to buy the service from the company can participate. In the former case, the participation rate is defined as the number of UKB participants divided by the number of people living in UK within 40-69 age group. However, the denominator is unclear in the latter case because it is difficult to identify exactly who is the potential customer. The term ‘implicit underlying target population’, in this case, points to this situation where the potential customers of the 23andMe is not identified.

As you will see in the following responses, we added two tables showing the magnitude of bias when running GWAS on a particular trait Y under sex-differential participation bias with low and high participation rate. When the participation rate is low, the degree of the bias is larger with other parameters (e.g. the log odds ratio of sex on participation) held fixed. Therefore, even when the size of the sample grows, without a well-defined target population in mind, it may enlarge the bias rather than reducing it. As the table was originally created while accounting the next comment, move on to the next section for more details.

We added the following sentences in the main article:

For example, the UK Biobank contacted the UK residents of age 40-69 in 2010. On the other hand, it is nearly impossible to know who the potential customer in case of commercial cohorts like 23andMe is. In the latter case, participation rate is extremely difficult to estimate as the denominator (the number of potential customers) is ill-defined.

3. The authors describe an important insight into the relationship between study participation rate and selection bias. However, I struggled to understand whether the authors were only addressing the scenario in which the overall participation rate is low, and how variation in the male/female imbalance given participation further affects the bias. Can the authors help the reader develop intuition for the following scenarios…
a) low participation rate overall - high sex imbalance
b) high participation rate overall - high sex imbalance
c) low participation rate overall - low sex imbalance
d) high participation rate overall - low sex imbalance

Response: We were able to produce some numerical examples based on the comment by the reviewer which revealed some interesting consequences of our theory that were invisible solely based on the formula. Using **Theorem 4** of the supplementary note, we calculated the direction and the magnitude of the bias under differing degrees and directions of both participation rate and sex imbalance.

What we found was very interesting in that the direction and the magnitude of the bias did not have a simple positive or negative correlation with the sex imbalance.

|  |  |
| --- | --- |
| Low participation rate | High participation rate |

We included the details of this analysis in **Section 6** of the **Supplementary Note**.

4. The participation rate discussed here and in referenced papers generally refers to the volunteer participation rate. Can the authors also comment on how these models hold in the context of an electronic health record/biobank study where the "participation rate" may reflect true differences in diagnosis? Furthermore, in a health care setting it is possible for the participation rate to reflect sex-biases in diagnostic practices as well as differences in health-care seeking behaviors between males and females (e.g., males visit the doctor less frequently and when they do visit they receive more diagnoses). It would be helpful for the authors to provide a comment on whether the underlying reason for the participation bias (which may be related to the disease, or related to other factors) matters at all for their proposed solution.

Response: As participation into a study can depend on various factors, the comment is a very natural and a relevant question. Our answer is that the example the reviewer suggested does not matter (if we understood correctly). Our mathematics does not depend on the specific mode of the effect of sex on participation. Therefore, the model is general enough to incorporate the case in which the reviewer described. We added the following sentences to the main article:

The theory is general enough to incorporate population-based studies like the UK Biobank and electronic health record (EHR)-based studies like DiscovEHR(11). This is because the mathematics behind the theory does not require any assumptions related to the specific mode of participation into the study. Unless the logit model of participation is not violated, it is likely that our theory will hold regardless of the study design.

Additionally, if relevant, we added an additional result regarding differing prevalence between sexes (**Theorem 5** in the **Supplementary note**). We consider a more general situation in which sex affects the true prevalence of the binary trait Y. We also included additional simulations showing the effect of true prevalence difference between sex in the **Supplementary Figures 3 and 6**.

5. Lastly, I would suggest the authors use "between sex" instead of "intersex" throughout the paper. The term "intersex" has both biological and clinical meaning, so used in this context is a bit confusing.

Response: Thank you for the comment. Indeed, intersex is used for certain type of medical and biological conditions that may be important to scientist and people who may have such conditions. We have changed the terminology across the manuscript so that the readers can avoid confusion.

In the interest of accountability in the review process, I choose to sign my reviews, Lea K. Davis.

Response: We are greatly thankful for extremely kind and constructive comments by Professor Lea K. Davis.

Reviewer #2: Review. A practical solution to correct for sex-differential participation bias: A theory- based Analysis

In this short report the authors provide a solution to correct for bias in genome-wide association studies due to sex-differential participation bias. Previous studies have shown the presence of such bias, but did not come with a solution. Here, a very logical and easily implementable solution is presented and tested.

Virtually all GWASs using population-based samples will be affected by participation bias, and sex-bias is likely to be an important bias. It's therefore highly relevant and valuable to address this bias and come to a solution. The proposed solution is no rocket science (as the authors notify themselves); it's simple and easy to implement (which is an important aspect). The paper should therefore be of broad interest to human geneticists and the proposed solution represents a significant advance over previous studies. The methodological details are thoroughly described (in the supplementary material) and code has been provided.

Response: We thank the reviewer for the great interest and kind comments on the work. The reviewer’ valuable comments led to a more comprehensible article that will help potential readers in the future.

The authors show a range of simulations that show the existence of sex-differential participation bias and supports their proposed solution. They may be able to extend their simulations/tests to traits with different prevalences (and prevalence differences between sexes) and a broader range of h2 (and h2 differences between sexes).

Response: The original article did not delt with different prevalence between sexes because previous simulation-based studies did not consider the situation. Hence, we developed and added **Theorem 5** (in the supplementary note) that deals with this situation.

Furthermore, as the reviewer pointed out, a more extensive simulation will make the article more persuasive. We expanded the range of parameters used in the simulation and the result can be found in **Supplementary Figures 1-6**.

h2 of participation used are 0, 0.1, 0.2, 0.3. The prevalence used are 0.1 and 0.5. For sex differential prevalence, the overall prevalence was set to 0.5 with prevalence in men and women were set to 0.1 and 0.3 respectively. Further details can be found in **Supplementary Note**’s appendix (as well as the code).

Furthermore, although I realize that the paper was submitted as a short report, the discussion may be broadened by discussing downsides of their solution (i.e. splitting samples can reduce statistical power), and by discussing other benefits of splitting samples (sex-differential loci can be detected).

Response: Power is a very important issue in GWAS and hence we provide both simulation and a theoretical perspective on the potential power loss of our solution.

We ran simulation comparing the -log(P-value) from GWAS from an unbiased sample and fixed-effects meta-analysis GWAS on a sample with sex-differential participation bias. As we see in the figure, distribution of P-values is nearly the same. The full figure can be found in **Supplementary Figure 7**.

In a theoretical viewpoint, this is predicted by combining Theorem 4 of our work and the work of Magi et al. (Genetic Epidemiology, 2010). Theorem 4 guarantees that the parameter (log odds ratio of the variant) being estimated is nearly the same in both sexes. Magi et al. shows that when the parameter being estimated is identical in both sexes, there is no loss of power. Hence, sex-stratified GWAS followed by fixed-effects meta-analysis is unlikely to suffer much from loss of power. We added the following sentences in the main article:

We show that this stratified approach does not result in loss of statistical power compared to combined GWAS in a representative sample without participation bias (**Supplementary Figure 7**).

Link for Magi et al.: https://onlinelibrary.wiley.com/doi/10.1002/gepi.20540

I also miss putting the approach in a broader perspective. Sex is one of the potential biases, but there are many others (e.g. age, SES). It's important to address sex-differential participation bias, but shouldn't the participation bias be addressed more generally, and how? This may be beyond the scope of the paper, but it should nevertheless be mentioned and discussed. In addition, there is another paper on participation bias (and its determinants) that may be relevant to refer to in the manuscript: <https://www.nature.com/articles/s41467-021-21073-y>.

Response: Collider bias is recently gaining a lot of interest so putting our work in a broader perspective will greatly help the readers. The suggestion of the reviewer is now reflected in the **Conclusion** section. The article in which the reviewer recommended was very helpful. The following has been included in the manuscript in the **Conclusion** section:

Our framework also provides insight into other forms of collider bias. The theory does not rely on a particular characteristic of sex except that it is binary so the argument can be applied to any binary variables that affect study participation. Furthermore, the proof can be naturally extended to any discrete variables with more than two values. In such a situation, given a low participation rate, the bias is likely to be small unless a genetic variant has an interaction with a variable that affects study participation, as shown in an earlier simulation study(6). If interaction exists, we can use a similar stratifying strategy for the multi-value variable to remove potential bias. In the future, further studies dealing with continuous variables are warranted(12), because continuous variables can also affect study participation.

Small remark:
Figure legends. "a loci" is incorrect, it should be "a locus" or "loci"

Response: Thank your pointing out our mistake. The typo is now resolved.

Reviewer #3: In the manuscript entitled "A practical solution to correct for sex-differential participation bias: A theory- based analysis", Lee and Han used theory and simulation to explain the biases in GWAS and follow-up analyses that use GWAS summary statistics due to sex-differential participation bias (i.e., the genetic effects on participation are sex-dependent). Based on their theory and simulation results, we concluded that if the participation rate is low, an efficient solution to correct participation biases is to perform a sex-stratified GWAS analysis. I have several concerns regarding the theoretical derivations and the simulation setting.

Response: Thank you for the thorough review going through our work carefully. The comments improved the manuscript significantly in several ways.

The derivations provided in the supplementary note are not easy to follow. For example, it is unclear what is likely to be the most realistic model in practice, and the authors attempted to use their theory to explain the three models proposed in Pirastu et al. without clearly stating what the models are.

Response: The supplementary have been re-written and the details of the models were filled to improve readability. We hope that the improved **Supplementary not**e has addressed your concerns.

I think the SNP effects on participation should be mediated through multiple phenotypes, and the number of phenotypes is likely to be large. However, I do not seem to find such a model in the authors' derivations. It would be interesting to see how the GWAS results for these intermediate traits would be biased by sex-differential participation. In part 4 of the supplementary note, the authors assumed a model where an SNP had a direct sex-dependent effect on participation and an indirect sex-dependent effect mediated through a phenotype. This model is less general than the model above, where all the SNP effects on participation are mediated through phenotypes.

Response: We agree with the comment, and we aimed to clarify this point in the revised version of our manuscript (particularly in the **Supplementary note**). In short, we tried to clarify that our model includes the ‘more general’ model that the reviewer pointed out. This is because the potential mediators of the gene effect are aggregated and represented in the arrow directly going from G to C. If the gene effect on participation C had been solely through Y, the direct arrow from G to C would have been needless. The description has been added to the supplementary note **Section 6**:

Note that Theorem 4 includes the scenario where the effect of variant Gj on participation C is pleiotropic (the effect is mediated by more than one trait). The theorem predicts the bias when running a GWAS on particular trait Y . Then the effect of variant Gj on C that is mediated by Y is depicted in Gj →Y →C and the rest of the effect that is not mediated by Y (but possibly by traits other than Y ) is depicted in Gj →C. If the effect of Gj on C was totally by Y , only Gj →Y →C would have been sufficient.

The authors may need to clarify why the bias in the SNP effect on the phenotype due to sex-differential participation bias is independent of the effect of the phenotype on participation in Theorem 4.

Response: We added a clarification of this point thanks to the comment. The effect of the phenotype on participation is included in the 1|yz (the probability is indexed by y which means the trait value is involved) term so the yc may not be visible on first sight. However, this becomes more explicit in the approximated version of the **Theorem 4**, namely **Corollary 4**¸where yc is involved explicitly. Additionally, while addressing the comment by the reviewer, we found an error in **Corollary 4** which led to a minor change in the theory section. We repeat our gratitude to the reviewer for pointing this out which eventually fixed a mathematical error.

The authors derived the theory for a binary phenotype. However, many phenotypes are quantitative. It would be nice if they could extend the theoretical derivations to quantitative phenotypes.

Response: We are sorry for the negative response. When starting this study, continuous phenotypes were also our primary target but we soon realized that the current proof technique could not result in a simple analytic solution as we have shown in the current paper for binary phenotypes. Nevertheless, future studies are required to better understand the effect of collider bias on continuous phenotypes.

We added the following sentences in the main article:

Our framework also provides insight into other forms of collider bias. The theory does not rely on a particular characteristic of sex except that it is binary so the argument can be applied to any binary variables that affect study participation. Furthermore, the proof can be naturally extended to any discrete variables with more than two values. In such a situation, given a low participation rate, the bias is likely to be small unless a genetic variant has an interaction with a variable that affects study participation, as shown in an earlier simulation study(6). If interaction exists, we can use a similar stratifying strategy for the multi-value variable to remove potential bias. In the future, further studies dealing with continuous variables are warranted(12), because continuous variables can also affect study participation.

Is it possible to predict by theory the genetic correlation between sex and a phenotype in the presence of participation bias?

Response: Yes. It is possible given the genetic correlation between study participation and a phenotype of interest with additional parameters that is required by the theory. By **Theorem 2**, gz=-gc1|1-1|0+zgc1-1|1 holds. Suppose that the effect size of a variant j on trait Y is gy. Then because the genetic correlation due to SNP is the correlation of betas, it is covgy,gz=-covgy,gc1|1-1|0+covgy,zgc1-1|1 =-1|1-1|0covgy,gc+1-1|1covgy,zgc

covgy,gc is the genetic covariance of pair (Y, C). covgy,zgc is the genetic covariance between Y and the effect size difference between men and women for study participation C. However, because C=0 is not observed, it is impossible to estimate gc and zgc from real data.

We added the above contents to **Supplementary Note Chapter 4.**

Under rare participation, it's unclear how Theorem 4 was simplified for model C. The authors may want to elaborate on the derivations on how Theorem 4 was simplified for the sex-stratified analysis.

Response: We agree to the reviewer’s comment that some parts of the derivation were too difficult for the readers to follow. We greatly expanded and added more details to the proofs and derivations across the manuscript and we hope this satisfies the reviewer.

The standard errors (SEs) of the estimates shown in Figure 1 are all too large. Since the SEs were observed from simulations, the authors can undoubtedly improve the precisions of the estimates by simulating a large sample and performing a larger number of simulations.

Response: Increasing the sample size improved the precision of figures. The figure now looks much better than before. Thank you for the comment.

I would add the MR result based on a meta-analysis of sex-stratified GWASs in Figure 1c.

Response: We added the result for fixed-effects meta-analysis to the Figure. Additionally, we ran multiple simulations with differing parameters to provide a more comprehensive view to the problem. These are listed in **Supplementary Figure 1-6**.

**2^nd^ round**

**Reviewer 1**

The authors have done a fantastic job of addressing my comments and suggestions!

**Reviewer 2**

The authors have satisfactorily addressed all my comments (as well as those from the other reviewers). I think this is a very good paper.

**Reviewer 3**

The authors have addressed all my previous concerns. I have no additional comments.
